# Microglial aryl hydrocarbon receptor enhances phagocytic function via SYK and promotes remyelination in the cuprizone mouse model of demyelination

**DOI:** 10.1186/s12974-023-02764-3

**Published:** 2023-03-25

**Authors:** Yumeng Wang, Jingxian Sun, Keying Zhu, Danjie Wang, Xiaoqiang Zhao, Hongyu Zhang, Shuai Wu, Yanqing Wang, Jun Wang

**Affiliations:** 1 Department of Integrative Medicine and Neurobiology, School of Basic Medical Science, Institutes of Integrative Medicine, State Key Laboratory of Medical Neurobiology and MOE Frontiers Center for Brain Science, Institutes of Brain Science, Shanghai Medical College, Fudan University, Shanghai, China; 2 Department of Clinical Neuroscience, Karolinska Institute, Center for Molecular Medicine, Karolinska University Hospital, Stockholm, Sweden; 3 Department of Neurology, Zhongshan Hospital, Fudan University, Shanghai, China

**Keywords:** AhR, Microglia, Phagocytosis, Remyelination, SYK

## Abstract

Multiple sclerosis (MS) is an inflammatory-mediated demyelinating disease of the central nervous system (CNS). Although studies have demonstrated that microglia facilitate remyelination in demyelinating diseases, the underlying mechanisms are still not fully characterized. We found that aryl hydrocarbon receptor (AhR), an environment sensor, was upregulated within the corpus callosum in the cuprizone model of CNS demyelination, and upregulated AhR was mainly confined to microglia. Deletion of AhR in adult microglia inhibited efficient remyelination. Transcriptome analysis using RNA-seq revealed that AhR-deficient microglia displayed impaired gene expression signatures associated with lysosome and phagocytotic pathways. Furthermore, AhR-deficient microglia showed impaired clearance of myelin debris and defected phagocytic capacity. Further investigation of target genes of AhR revealed that spleen tyrosine kinase (SYK) is the downstream effector of AhR and mediated the phagocytic capacity of microglia. Additionally, AhR deficiency in microglia aggravated CNS inflammation during demyelination. Altogether, our study highlights an essential role for AhR in microglial phagocytic function and suggests the therapeutic potential of AhR in demyelinating diseases.

**Graphical Abstract:**

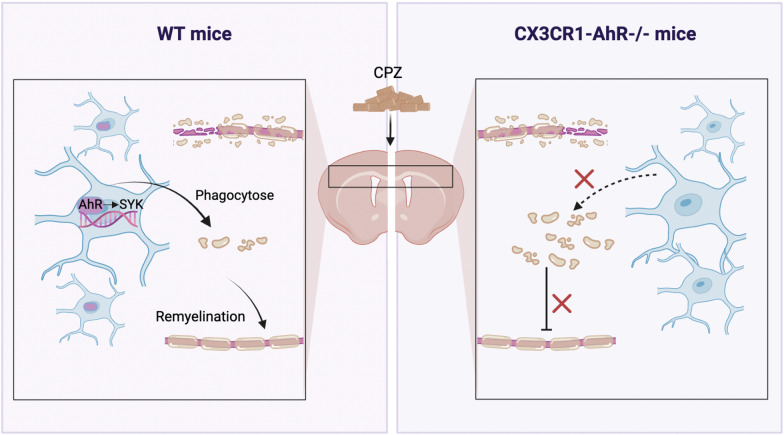

**Supplementary Information:**

The online version contains supplementary material available at 10.1186/s12974-023-02764-3.

## Highlights

AhR regulates remyelination following cuprizone-induced demyelination.

Deletion of AhR in adult microglia restrains efficient remyelination.

Microglial AhR deficiency causes myelin debris accumulation and impairs phagocytic function.

Microglial AhR mediates phagocytosis of microglia via SYK.

**Supplementary Information:**

The online version contains supplementary material available at 10.1186/s12974-023-02764-3.

## Introduction

Microglia are the resident immune cells of the central nervous system (CNS), distributed in the parenchyma of the brain and spinal cord [1]. Microglia play an essential role in CNS development and tissue homeostasis. Microglia modulate processes including synaptic pruning, myelogenesis, oligodendrocyte differentiation, and neurotrophic support [2]. Microglia rapidly transform into reactive states manifested by morphological changes, increased cytokine secretion, and enhanced phagocytic ability in multiple pathological states [3]. Furthermore, in neurodegenerative diseases such as Alzheimer's disease (AD), multiple sclerosis (MS), and Parkinson’s disease (PD), microglia play a dual role in neuroinflammation and neuroprotection [4]. In recent years, studies have suggested that one of the major roles of microglia in MS is the regenerative functions in supporting remyelination [5, 6]. However, the underlying mechanisms of microglia in remyelination remain not fully characterized.

At present, the pathogenesis of multiple sclerosis remains unclear. It is generally believed that MS is driven by autoimmune attacking myelin sheath leads to demyelinating and even secondary axonal injury, affecting millions of people worldwide [7]. In addition to genetic variation, key contributors to MS risk include environmental factors and lifestyle [8]. Current treatments of MS alleviate symptoms to some extent but have a limited effect in alleviating neurological deficits and dysfunction [9, 10]. No effective treatment specifically targeting myelin regeneration has been applied clinically yet. Myelin is a dense structure synthesized by oligodendrocytes wrapped spirally around axons, conferring protections to the underlying axons. Most importantly, myelin sheath is conducive to saltatory conduction and rapid conduction of the action potential along neurons [11–13]. Therefore, it is crucial to identify potential targets for promoting myelin regeneration after demyelination.

Aryl hydrocarbon receptor (AhR) is a cytoplasmic receptor and transcription factor belonging to the basic helix-loop-helix (bHLH) superfamily. AhR functions as an environmental sensor, integrating environmental and dietary factors to control complex transcriptional procedures in a ligand-specific manner [14]. AhR affects a variety of physiological and pathological processes by regulating the expression of downstream target genes. AhR regulates the differentiation and function of a variety of immune cells and mediates the process of inflammation regression [15, 16]. In addition, AhR is ubiquitously expressed in mammalian CNS [17]. A functional study has shown that AhR is involved in regulating the proliferation and differentiation of neural stem cells [18]. AhR knockout mice suffer from impaired neurogenesis and cognitive dysfunction in the hippocampus [19].

AhR drives anti-inflammatory function in peripheral immune cells and CNS resident cells to alleviate inflammatory diseases [15]. Indeed, AhR is aberrantly expressed in MS lesion areas [20]. Recent studies have shown that AhR endogenous agonist levels are significantly lower in MS patients compared to healthy controls and lower AhR agonist levels are correlated with neurological dysfunction and disease severity [21, 22], implying that AhR may play an essential role in the pathogenesis of MS. In experimental autoimmune encephalitis (EAE), an animal model of MS, administering agonists to activate AhR suppresses disease progression [20, 23, 24], whereas inhibiting AhR and its immunosuppressive function promotes immune responses and exacerbates EAE [25]. Moreover, microglial AhR modulates the pathogenic activity of astrocytes and CNS inflammation in the EAE model [26]. However, the roles and mechanisms of AhR in demyelination and remyelination in MS have not been fully illustrated.

In this study, we used a cuprizone (CPZ)-induced demyelinating animal model. Cuprizone exclusively induces oligodendrocyte death, resulting in demyelination in the corpus callosum, hippocampus, cerebellum and other brain areas, with the corpus callosum being the most severely affected [27]. Demyelination is primary, accompanied by the degeneration of oligodendrocytes and the activation of microglia and astrocytes. Remyelination begins within one week of resuming a normal diet, and the mechanism of remyelination is similar to spiral wrapping during development [28]. The cuprizone model considerably facilitates our understanding of dissimilar aspects of MS pathology compared to the EAE model. EAE model exhibits the pathological features and the complex immune responses in MS, whereas cuprizone model allows researchers to investigate mechanisms underlying demyelination and remyelination without the influence of adaptive immune system [29, 30]. We identified that microglial AhR was activated in cuprizone model. AhR deficiency in microglia inhibited efficient remyelination, while the administration of an AhR agonist promoted remyelination. Further investigation revealed that microglial AhR enhanced remyelination by regulating the expression of spleen tyrosine kinase (SYK), a phagocytosis-related gene, to facilitate the clearance of myelin debris by microglia. Moreover, we discovered that microglial AhR deficiency exacerbated CNS inflammation.

## Results

### Cuprizone increases AhR expression and transcriptional activity in the corpus callosum

**Fig. 1:**
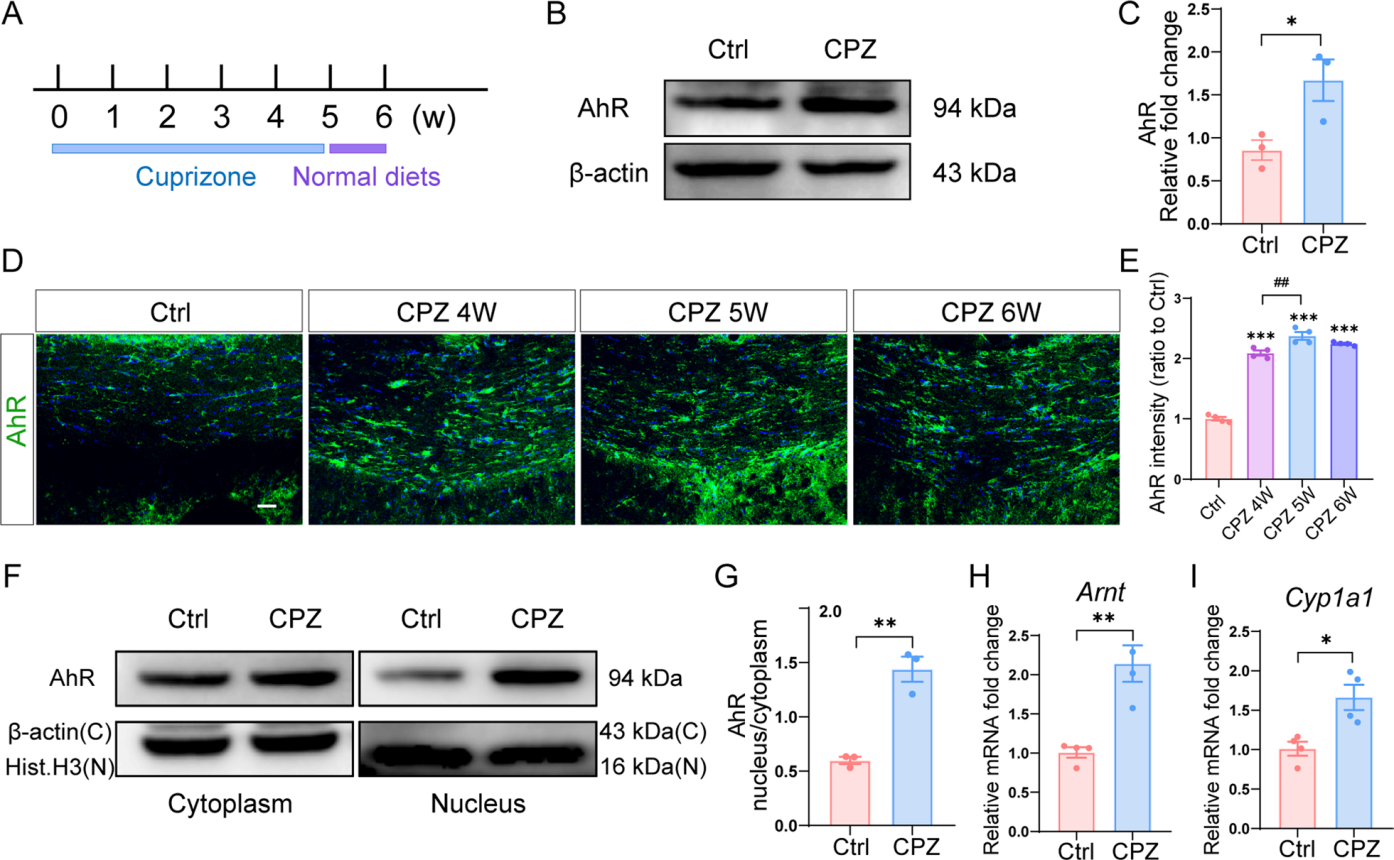
AhR expression and transcriptional activity are upregulated in the corpus callosum during demyelination. **A** Schematic illustration of the cuprizone demyelinating model. **B** Representative western blot analysis of AhR protein level in the corpus callosum of control mice and cuprizone-treated mice (in the context of the end of week 6 in the cuprizone model). 30 μg protein was loaded per well. **C** The quantification of AhR protein expression in **B** (n = 3 mice per group). Data are shown as mean ± SEM and analyzed by unpaired two-tailed t-test. **D** Representative confocal immunofluorescent images displaying AhR expression in the corpus callosum at the end of week 4, week 5 and week 6 of cuprizone model. Scale bar: 40 μm. **E** The quantification of AhR fluorescence intensity in **D** (n = 4 mice per group). Data are shown as mean ± SEM and analyzed by one-way ANOVA with Tukey’s multiple comparisons test. **F** AhR protein level were assessed in the cytoplasm and nucleus of control mice and cuprizone-treated mice (at the end of week 6 of cuprizone model) via Western blot. 30 μg protein was loaded per well. **G** The quantification of AhR protein expression in **F**. (n = 3 mice per group). Data are shown as mean ± SEM and analyzed by unpaired two-tailed t-test. **H**, **I** mRNA expressions of *Arnt* and *Cyp1a1* in the corpus callosum of control mice and cuprizone-treated mice (at 6 weeks post cuprizone) were evaluated by qRT-PCR (n = 4 mice per group). Data are shown as mean ± SEM and analyzed by unpaired two-tailed t-test. *p < 0.05, **p < 0.01, ***p < 0.001, ##p < 0.01

To investigate whether AhR is involved in CNS remyelination, we first set up a demyelinating animal model by feeding mice a diet containing 0.3% cuprizone for 5 weeks. Afterward, normal chow feeding was resumed at week 6 for a week for early spontaneous myelin repair (Fig. 1A). As assessed by western blot, the expression of AhR in the corpus callosum was significantly upregulated in cuprizone-treated mice (Fig. 1B and C). We examined AhR expression at the end of week 4, week 5, and week 6 of the cuprizone model by immunofluorescence staining, and AhR expression was highest at the end of week 5 (Fig. 1D and E). In addition, cuprizone administration induced AhR activation as indicated by an increased AhR cytoplasm-to-nuclei translocation (Fig. 1F and G). Accordingly, the expression of the AhR nuclear transporter *Arnt* and AhR target gene *Cyp1a1* was also increased in the cuprizone model (Fig. 1H and I), indicating that AhR transcriptional activity was enhanced. These data suggested that AhR is involved during cuprizone-induced demyelination.

### AhR regulates remyelination following cuprizone-induced demyelination

**Fig. 2:**
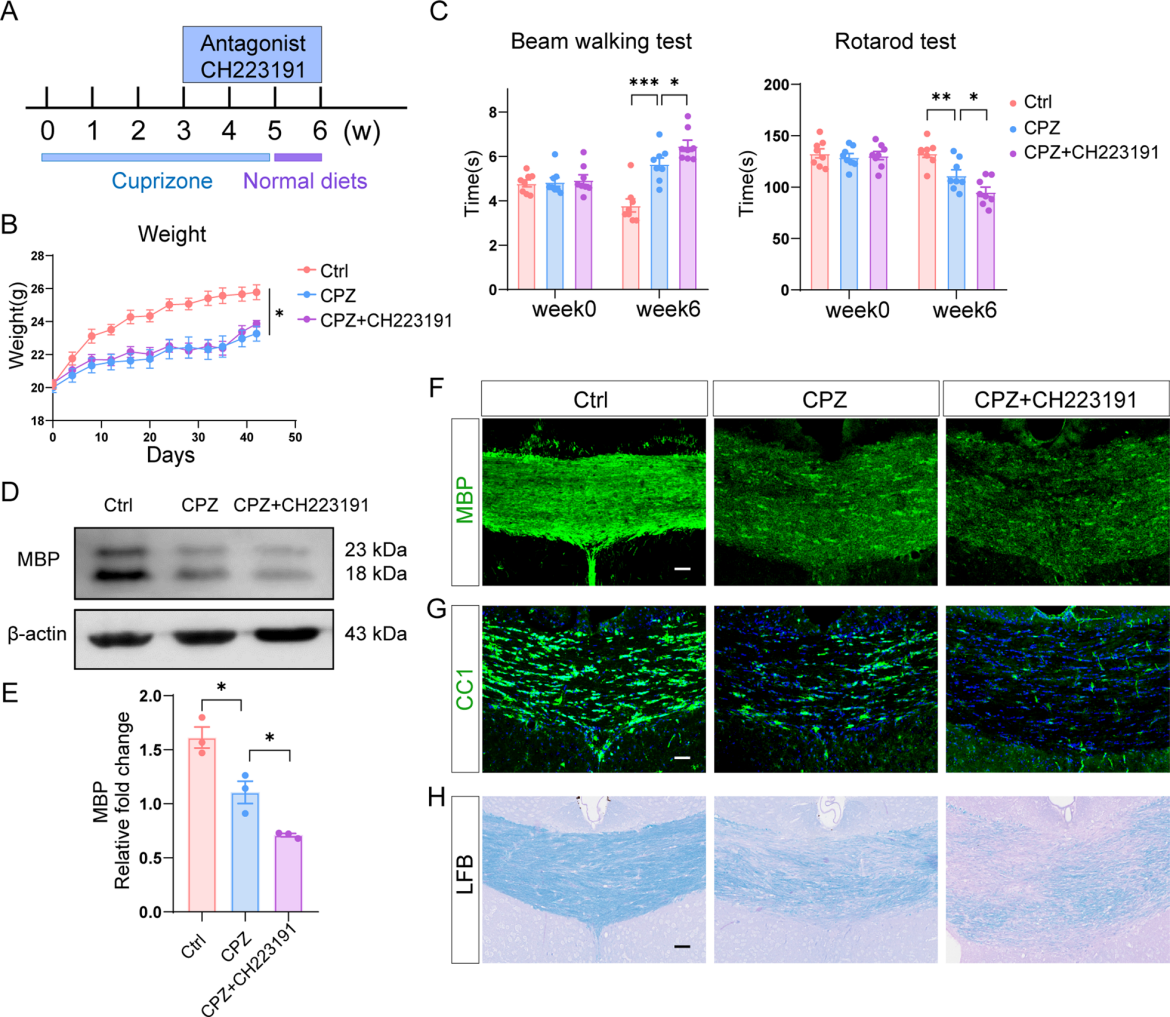
Inhibition of AhR restrains remyelination in cuprizone model. **A** Time course of CH223191 administration in cuprizone model. **B** Body weight of control mice, cuprizone-treated mice and mice with CH223191 administration (n = 8 mice per group). Data are shown as mean ± SEM and analyzed by two-way ANOVA with Tukey’s multiple comparisons test. **C** Beam walking test and rotarod test evaluating motor coordinative function were performed in 3 experimental groups (n = 8 mice per group). Data are shown as mean ± SEM and analyzed by two-way ANOVA with Tukey’s multiple comparisons test. **D** Western blot analysis of MBP protein level in the corpus callosum of control mice, mice in cuprizone model and mice with CH223191 administration. 25 μg protein was loaded per well. **E** The quantification of MBP protein expression in **D** (n = 3 mice per group). Data are shown as mean ± SEM and analyzed by one-way ANOVA with Tukey’s multiple comparisons test. **F** Representative confocal immunofluorescent images displaying MBP expression in the corpus callosum of 3 experimental groups. Scale bar: 40 μm. **G** Representative confocal immunofluorescent images of CC1 staining in the corpus callosum of 3 experimental groups. Scale bar: 40 μm. **H** Representative LFB stained images in the corpus callosum of control mice, mice in cuprizone model and mice with CH223191 administration. Scale bar: 40 μm. *p < 0.05, **p < 0.01, ***p < 0.001. The results of this figure are acquired at the end of week 6 of the cuprizone model

Since exposure to cuprizone increases AhR expression and transcriptional activity, we then performed loss-of-function experiments by inhibiting AhR activity to further explore the role of AhR in demyelination. AhR function was suppressed by the antagonist CH223191 at a dose of 10 mg/kg for 3 weeks (Fig. 2A). The body weight of mice fed with cuprizone food was lower than that of control mice (Fig. 2B). Behavioral tests including beam walking test and rotarod test were performed to evaluate the motor coordinative function of mice. Cuprizone-treated mice took longer time to pass through the beam and stay less time on the rotarod than control mice, indicating that the motor coordinative function was impaired following cuprizone administration (Fig. 2C). The AhR antagonist CH223191 further aggravated the motor coordinative dysfunction in cuprizone-treated mice (Fig. 2C).

Myelin basic protein (MBP) maintains the structure and function of myelin sheath, and its expression represents the degree of myelination. As expected, MBP expression in the corpus callosum was significantly decreased following cuprizone treatment and was further reduced following CH223191 administration (Fig. 2D–F). In addition, we observed a significant decrease in the number of CC1^+^ mature oligodendrocytes after CH223191 treatment (Fig. 2G). The use of the Luxol Fast Blue (LFB) staining, which labels myelin sheath, revealed a more pronounced loss of myelin sheath after CH223191 administration (Fig. 2H).

We further performed gain-of-function validation exploring whether the activation of AhR by the agonist indoxyl-3-sulfate (I3S) had a beneficial effect in the cuprizone model (Additional file 1A). I3S is an endogenous agonist and a microbial metabolite of tryptophan [20]. I3S had no effect on body weight in mice treated with or without cuprizone (Additional file 1B). Behavioral tests showed that I3S significantly improved the motor coordinative function in cuprizone-treated mice (Additional file 1C). Moreover, I3S increased MBP expression and increased the number of mature oligodendrocytes in the corpus callosum after demyelination (Additional file 1D–G). We also administered I3S to control mice to explore whether I3S affected motor coordinative function and myelin-related protein levels in the absence of cuprizone. The findings revealed that I3S had no effect on motor coordinative function (Additional file 1C). Furthermore, I3S treatment had no effect on MBP expression or the number of mature oligodendrocytes in control mice (Additional file 1D–G).

Taken together, these data suggest that AhR is important in regulating remyelination following cuprizone-induced demyelination.

### Microglial AhR deficiency restrains remyelination

**Fig. 3:**
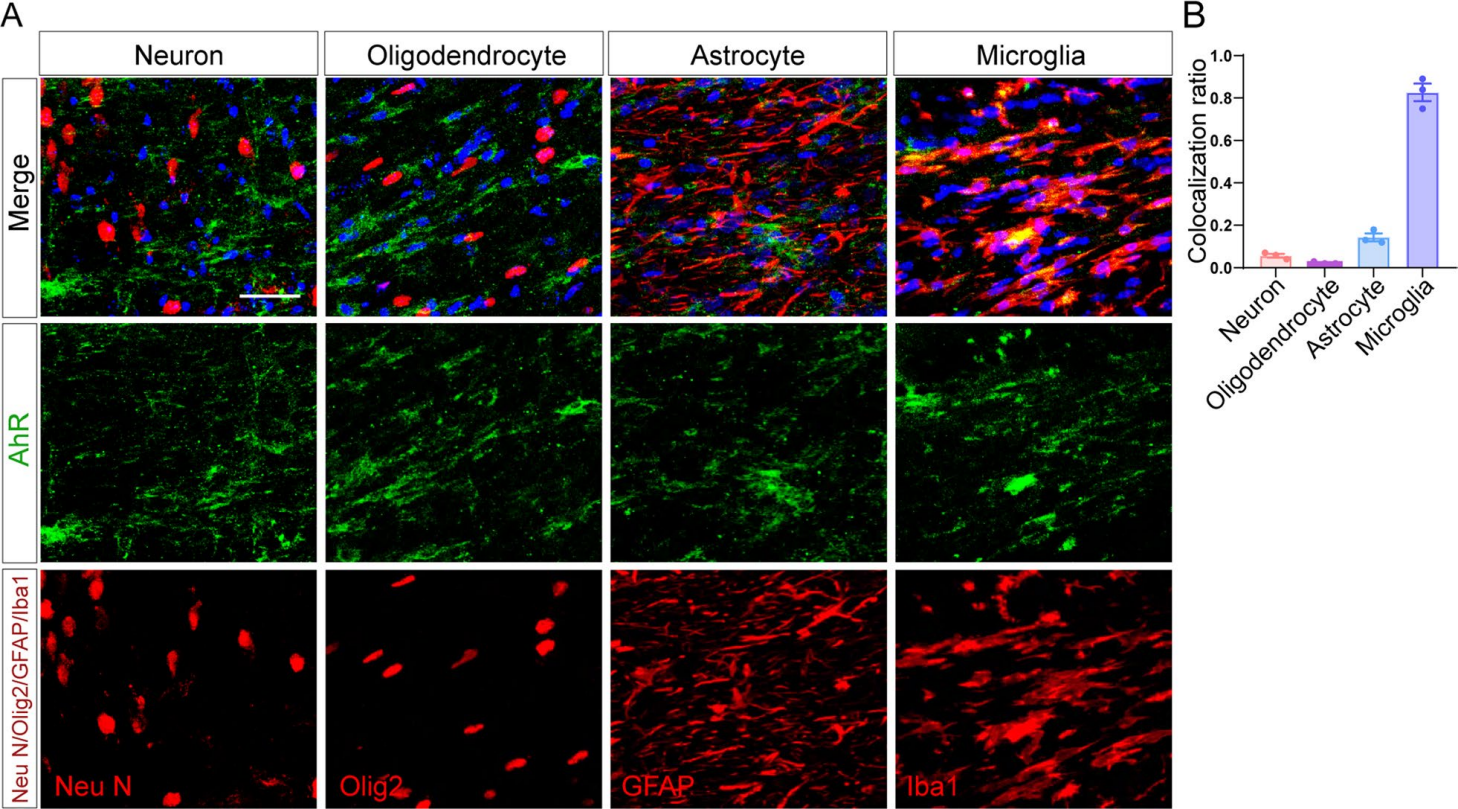
AhR expression in the corpus callosum of cuprizone model is mainly confined to microglia. **A** Representative confocal immunofluorescent images showing co-staining of AhR (green) with NeuN, Olig2, GFAP and Iba1 (red) in the corpus callosum of cuprizone-treated mice (after a 5-week cuprizone administration). Scale bar: 40 μm. **B** The quantification for colocalization ratio of AhR and cell markers in (J) (n = 3 mice per group). Data are shown as mean ± SEM

We next wondered which cell type in the CNS executes the function of AhR in the corpus callosum. By employing double immunofluorescence staining, we confirmed that AhR is mainly expressed in Iba1^+^ cells which, in the context of the end of week 5 in the cuprizone model, are mainly microglia (Fig. 3A and B). NeuN^+^ neurons, Olig2^+^ cells of the oligodendrocyte lineage, and GFAP^+^ astrocytes showed much lowered colocalization with AhR as compared to that of the Iba1^+^ cells. To address the role of AhR in microglia, we thus bred microglia-inducible Cre-ERT2 (*Cx3cr1*^CreERT2^) mice with *Ahr*^fl/fl^ and generated *Cx3cr1*^CreERT2^*Ahr*^fl/fl^ mice in which tamoxifen administration results in the activation of Cre recombinase and *Ahr* knockout in the *Cx3cr1*-expressing microglia. After 5 weeks of cuprizone treatment, we validated that AhR remained minimally expressed and none of AhR was colocalized with microglia in CX3CR1-AhR^−/−^ mice (Additional file 2A). The transcriptional level of the AhR target gene *Cyp1a1* in the corpus callosum was also decreased (Additional file 2B).

**Fig. 4:**
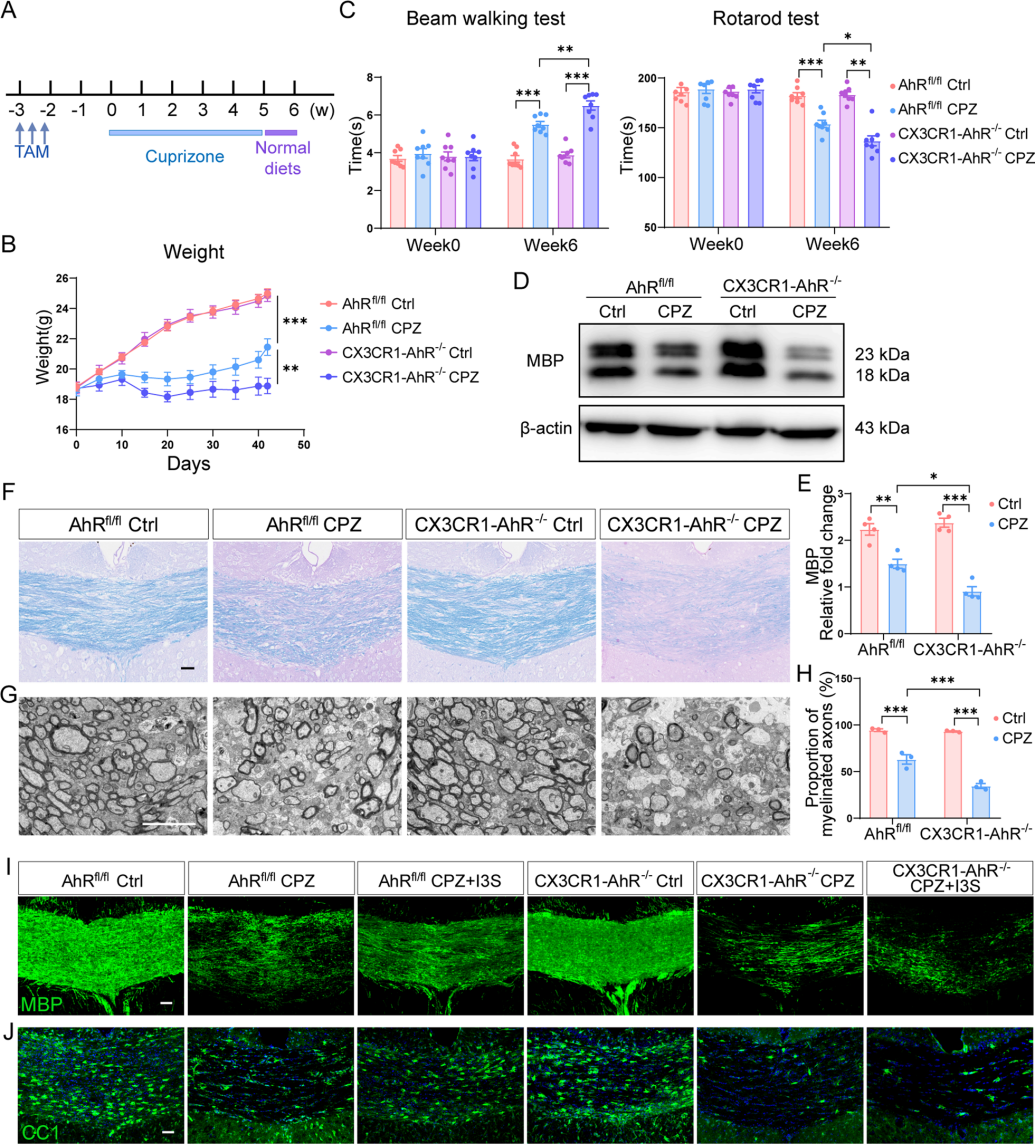
AhR deficiency in microglia inhibits remyelination. **A** Schematic of tamoxifen treatment and experimental timeline. **B** Body weight of AhR^fl/fl^ mice and CX3CR1-AhR^−/−^ mice during demyelination (n = 8 mice per group). Data are shown as mean ± SEM and analyzed by two-way ANOVA with Tukey’s multiple comparisons test. **C** Beam walking test and rotarod test evaluating motor coordinative function were performed in AhR^fl/fl^ mice and CX3CR1-AhR^−/−^ mice (n = 8 mice per group). Data are shown as mean ± SEM and analyzed by two-way ANOVA with Tukey’s multiple comparisons test. **D** Representative western blot analysis of MBP protein level in the corpus callosum of AhR^fl/fl^ mice and CX3CR1-AhR^−/−^ mice. 25 μg protein was loaded per well. **E** The quantification of MBP protein expression in **D** (n = 4 mice per group). Data are shown as mean ± SEM and analyzed by two-way ANOVA with Tukey’s multiple comparisons test. **F** Representative LFB stained images in the corpus callosum of AhR^fl/fl^ mice and CX3CR1-AhR^−/−^ mice at the end of week 6 of the cuprizone model. Scale bar: 40 μm. **G** Representative EM images of the ultrastructure in the corpus callosum of AhR^fl/fl^ mice and CX3CR1-AhR^−/−^ mice at the end of week 6 of the cuprizone model. Scale bar: 5 μm. **H** The quantification for proportion of myelinated axons in **G** (n = 3 mice per group). Data are shown as mean ± SEM and analyzed by two-way ANOVA with Tukey’s multiple comparisons test. **I** Representative confocal immunofluorescent images displaying MBP expression in the corpus callosum of AhR^fl/fl^ mice and CX3CR1-AhR^−/−^ mice with or without I3S treatment. Scale bar: 40 μm. **J** Representative confocal immunofluorescent images of CC1 staining in the corpus callosum of AhR^fl/fl^ mice and CX3CR1-AhR^−/−^ mice with or without I3S administration. Scale bar: 40 μm. *p < 0.05, **p < 0.01, ***p < 0.001. The results of this figure are acquired in the context of the end of week 6 in the cuprizone model

CX3CR1-AhR^−/−^ mice and AhR^fl/fl^ mice (littermate control mice) were subjected to cuprizone treatment 3 weeks after tamoxifen administration (Fig. 4A). The body weight of the CX3CR1-AhR^−/−^ mice was lower compared with the littermate control mice during demyelination (Fig. 4B). Microglial AhR deletion did not induce motor coordinative dysfunction under normal circumstances but further dampened the behavioral performance of mice under the demyelinating condition (Fig. 4C). The cuprizone model is a long-term model that includes five weeks of injury and one week of early spontaneous remyelination (Fig. 4A). Immunofluorescent staining of MBP in the corpus callosum revealed that the protein level of MBP was decreased in all mice after 5 weeks of cuprizone treatment, with no significant difference between the two genotypes (Additional file 3A and B), indicating that AhR deficiency had no effect on demyelination. However, remyelination was delayed in CX3CR1-AhR^−/−^ mice as characterized by decreased MBP expression using Western blotting and more pronounced loss of myelin sheath using LFB staining at the end of week 6 of cuprizone model (Fig. 4D–F). In parallel with reduced expression of myelin-related protein, transmission electron microscopy (TEM) also revealed a reduced proportion of myelinated axons in the corpus callosum of CX3CR1-AhR^−/−^ mice as compared to that of the AhR^fl/fl^ mice after cuprizone administration (Fig. 4G and H). In addition, administration of the AhR agonist I3S reversed the expression of MBP and the number of CC1 + mature oligodendrocytes in the corpus callosum of AhR^fl/fl^ mice but failed in CX3CR1-AhR^−/−^ mice (Fig. 4I and J), indicating that microglial AhR is required to mediate the pro-remyelination effect of I3S. These results collectively show that microglial AhR function determines the efficiency of remyelination.

### AhR deficiency in microglia induces a distinct transcriptional profile in demyelinating condition

**Fig. 5:**
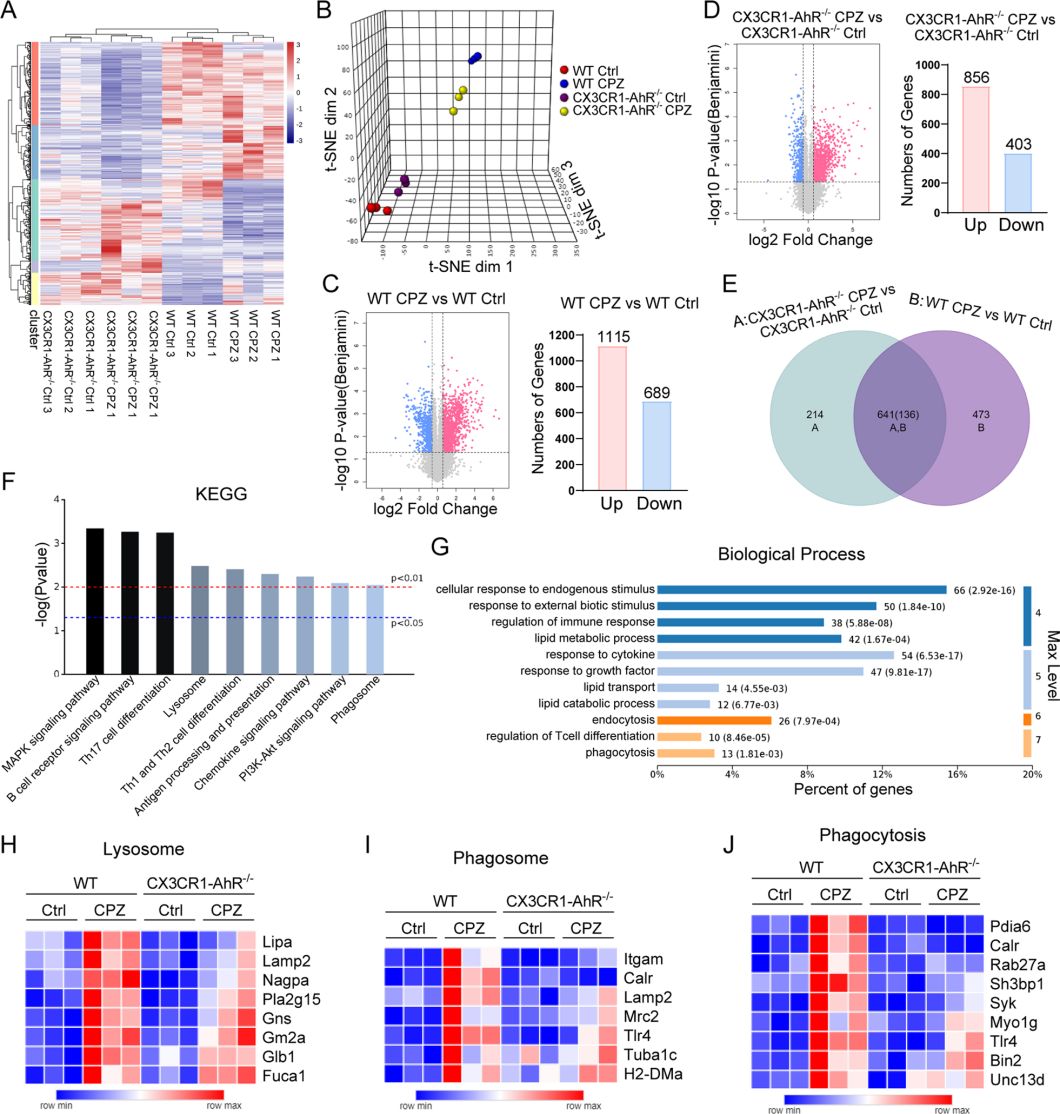
Microglial AhR deficiency induces a distinct transcriptional profile in demyelinating condition. **A** Heatmap of differential gene transcripts in WT mice and CX3CR1-AhR^−/−^ mice after 5 weeks of cuprizone administration (n = 3 mice per group; fold change > 1.5, adjusted p < 0.05). **B** tSNE projection of 4 experimental groups. **C** Volcano plot depicting upregulated and downregulated genes and number of genes upregulated and downregulated in WT mice owing to cuprizone treatment (n = 3 mice per group; fold change > 1.5, adjusted p < 0.05). **D** Volcano plot depicting upregulated and downregulated genes and number of genes upregulated and downregulated in CX3CR1-AhR^−/−^ mice owing to cuprizone treatment (n = 3 mice per group; fold change > 1.5, adjusted p < 0.05). **E** Venn Diagram of differential genes unique to WT mice and CX3CR1-AhR^−/−^ mice (n = 3 mice per group; fold change > 1.5, adjusted p < 0.05). **F** KEGG pathway analysis of 473 upregulated genes unique to WT mice after demyelination. **G** GO term enrichment analysis of 473 upregulated genes unique to WT mice after demyelination. **H–J** Heatmap of differential transcripts associated with lysosome, phagosome and phagocytosis (n = 3 mice per group; fold change > 1.5, adjusted p < 0.05)

To gain insights into the underlying mechanisms of microglial AhR function, we used RNA-seq to compare the transcriptional programming in the corpus callosum between the wild-type (WT) and CX3CR1-AhR^−/−^ mice following cuprizone-induced demyelination. The transcriptional profiling of the 4 different groups (WT Ctrl, WT CPZ, CX3CR1-AhR^−/−^ Ctrl, and CX3CR1-AhR^−/−^ CPZ) was distinct from each other, and samples within the same groups were well-clustered together (Fig. 5A and B). Cuprizone administration led to transcriptional changes in both the WT and CX3CR1-AhR^−/−^ mice as compared to the control mice, whereas the transcriptional responses to cuprizone treatment also differed between the WT and CX3CR1-AhR^−/−^ mice. After demyelination, 1804 gene transcripts in WT mice were significantly changed compared to the control group, and 1259 gene transcripts in CX3CR1-AhR^−/−^ mice (fold change > 1.5, adjusted p < 0.05) (Fig. 5C and D). The majority of gene transcripts were upregulated (Fig. 5C and D). Among the upregulated genes, we identified 473 that were unique to WT mice and 214 that were unique to CX3CR1-AhR^−/−^ mice (Fig. 5E). According to KEGG pathway analysis, these 473 upregulated genes were enriched in classical signaling pathways such as MAPK signaling pathway and PI3K-Akt signaling pathway, as well as the lysosome and phagosome pathways (Fig. 5F). These genes were also found to be involved in endocytosis and phagocytosis, as well as lipid transport and lipid catabolic process, according to GO term enrichment analysis (Fig. 5G). Phagocytosis and subsequent lipid metabolic process contribute to removing myelin debris, which is required for efficient remyelination. Previous studies reveal that myelin injury increased the expression of genes involved in phagocytosis and lysosome pathways in microglia [5, 31]. The significant upregulation of genes related to lysosome and phagosome after demyelination was consistent with the previous studies [5, 31] (Fig. 5H–J). Compared to the WT-CPZ group, microglial AhR deficiency following cuprizone treatment led to insufficient induction of genes related to lysosome, phagosome, and phagocytosis, indicating that certain functions of microglia, such as protein degradation and debris clearance, may be impaired (Fig. 5H–J).

### Microglial AhR deficiency causes myelin debris accumulation and impaired phagocytic function

Immunofluorescence staining revealed that microglial AhR deficiency increased the percentage of positive staining of the microglia marker Iba1 in the corpus callosum of the cuprizone model (Additional file 4A and B). We then performed skeleton analysis of microglia in AhR^fl/fl^ mice and CX3CR1-AhR^−/−^ mice with or without cuprizone treatment (Additional file 4A). Microglia in the corpus callosum of AhR^fl/fl^ control mice and CX3CR1-AhR^−/−^ control mice displayed proper morphology with standard ramified processes, whereas microglia in AhR^fl/fl^ CPZ mice had more process endpoints and longer process length (Additional file 4A, C and D). Moreover, both the process endpoints and the process length of microglia in CX3CR1-AhR^−/−^ CPZ mice were much higher than AhR^fl/fl^ CPZ mice (Additional file 4A, C and D). These findings show that microglial AhR deficiency altered microglial morphological changes in the cuprizone model, implying that AhR may influence microglia function.

**Fig. 6:**
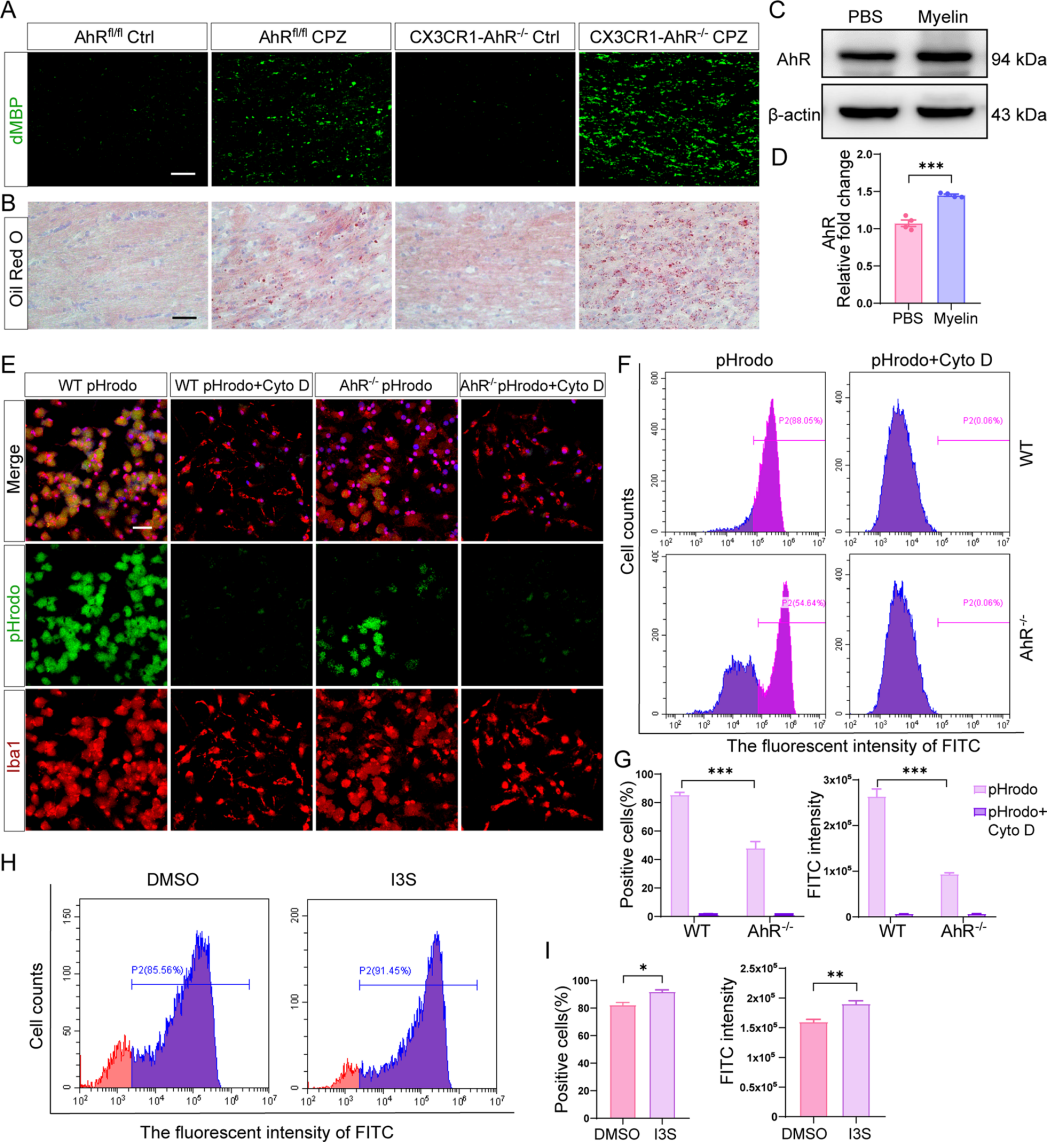
Microglial AhR deletion affects the clearance of myelin debris and phagocytic capacity of microglia. **A** Representative confocal immunofluorescent images of degraded MBP (dMBP) staining in the corpus callosum of AhR^fl/fl^ mice and CX3CR1-AhR^−/−^ mice after a 5-week cuprizone administration. Scale bar: 40 μm. **B** Representative Oil red O stained images in the corpus callosum of AhR^fl/fl^ mice and CX3CR1-AhR^−/−^ mice after 5 weeks of cuprizone treatment. Scale bar: 50 μm. **C** Western blot analysis of AhR protein level in WT primary microglia treated with PBS or purified myelin. 30 μg protein was loaded per well. **D** The quantification of AhR protein expression in **C** (n = 4 biological replicates). Data are shown as mean ± SEM and analyzed by unpaired two-tailed t-test. **E** Representative confocal immunofluorescent images displaying the uptake of pHrodo green bioparticles by WT primary microglia and AhR^−/−^ primary microglia with or without cytochalasin D treatment. Scale bar: 20 μm. **F** Flow cytometry analysis of the phagocytosed pHrodo green bioparticles by WT primary microglia and AhR^−/−^ primary microglia with or without cytochalasin D treatment (n = 3 biological replicates). **G** The quantification of positive cells engulfing pHrodo green bioparticles and FITC fluorescent intensity in 4 experimental groups (n = 3 biological replicates). Data are shown as mean ± SEM and analyzed by two-way ANOVA with Tukey’s multiple comparisons test. **H** Flow cytometry analysis of the phagocytosed pHrodo bioparticles by WT primary microglia treated with DMSO or agonist I3S. **I** The quantification of positive cells engulfing pHrodo green bioparticles and FITC fluorescent intensity in 2 experimental groups (n = 3 biological replicates). Data are shown as mean ± SEM and analyzed by unpaired two-tailed t-test. *p < 0.05, **p < 0.01, ***p < 0.001

Given that the clearance of myelin debris by microglia is critical for efficient remyelination following demyelinating injury, and microglial AhR deletion inhibited phagocytosis pathway (Fig. 5I and J), we hypothesized that AhR might affect remyelination by regulating the phagocytic uptake of myelin debris by microglia. To test this hypothesis, we first looked for the myelin debris in the corpus callosum using immunofluorescence staining for degraded MBP (dMBP). We found excessive myelin debris in AhR conditional knockout mice versus littermate control mice after a 5-week cuprizone administration (Fig. 6A). Oil-Red-O staining which labels lipid droplets in myelin debris also proved that lipid deposition in the corpus callosum was significantly increased in AhR conditional knockout mice (Fig. 6B). Collectively, microglial AhR deficiency affects the clearance of myelin debris by microglia.

We further examined whether AhR affected the phagocytic function of microglia. From our in vitro experiments, we noted that AhR expression was significantly upregulated in WT primary microglia after incubation with purified myelin for 16 h (Fig. 6C and D). pHrodo Green *E. coli* BioParticles, which are pH-sensitive dye conjugates that do not fluoresce outside cells, but fluoresce bright green when uptaken into phagosomes where the pH environment is acidic, were also employed to detect the phagocytic capacity of primary microglia. The fluorescent intensity of pHrodo, therefore, reflects the phagocytic ability of cells. We included a cytochalasin D treatment group as a negative control as cytochalasin D inhibits actin polymerization. Immunofluorescence staining identified a significantly reduced uptake of pHrodo green bioparticles in AhR knockout microglia as compared to the WT microglia (Fig. 6E), indicating a marked defect in phagocytic ability. In addition, flow cytometry also proved a decreased phagocytic capacity of AhR knockout microglia (Fig. 6F), as evidenced by a lower percentage of positive cells engulfing pHrodo green bioparticles and a lower FITC fluorescent intensity (Fig. 6G). Moreover, flow cytometry results showed that after administration of agonist I3S, the percentage of positive cells engulfing pHrodo green bioparticles was increased and the FITC fluorescent intensity was upregulated (Fig. 6H and I), indicating that AhR agonist I3S improved phagocytic function of WT primary microglia. Altogether, these findings demonstrated that microglial AhR deletion impairs phagocytic ability, resulting in myelin debris accumulation in the corpus callosum, and thereby may inhibit efficient remyelination.

We cultured mouse primary microglia and in vitro studies showed that administration of AhR agonist I3S at a dose of 10 μM did not affect microglial survival as analyzed via CellTiter-Glo assay (Additional file 5A). Both scratch-wound assay and transwell assay confirmed that AhR activation did not affect microglial migration (Additional file 5B–D).

### SYK induced by AhR is essential for phagocytosis of microglia

**Fig. 7:**
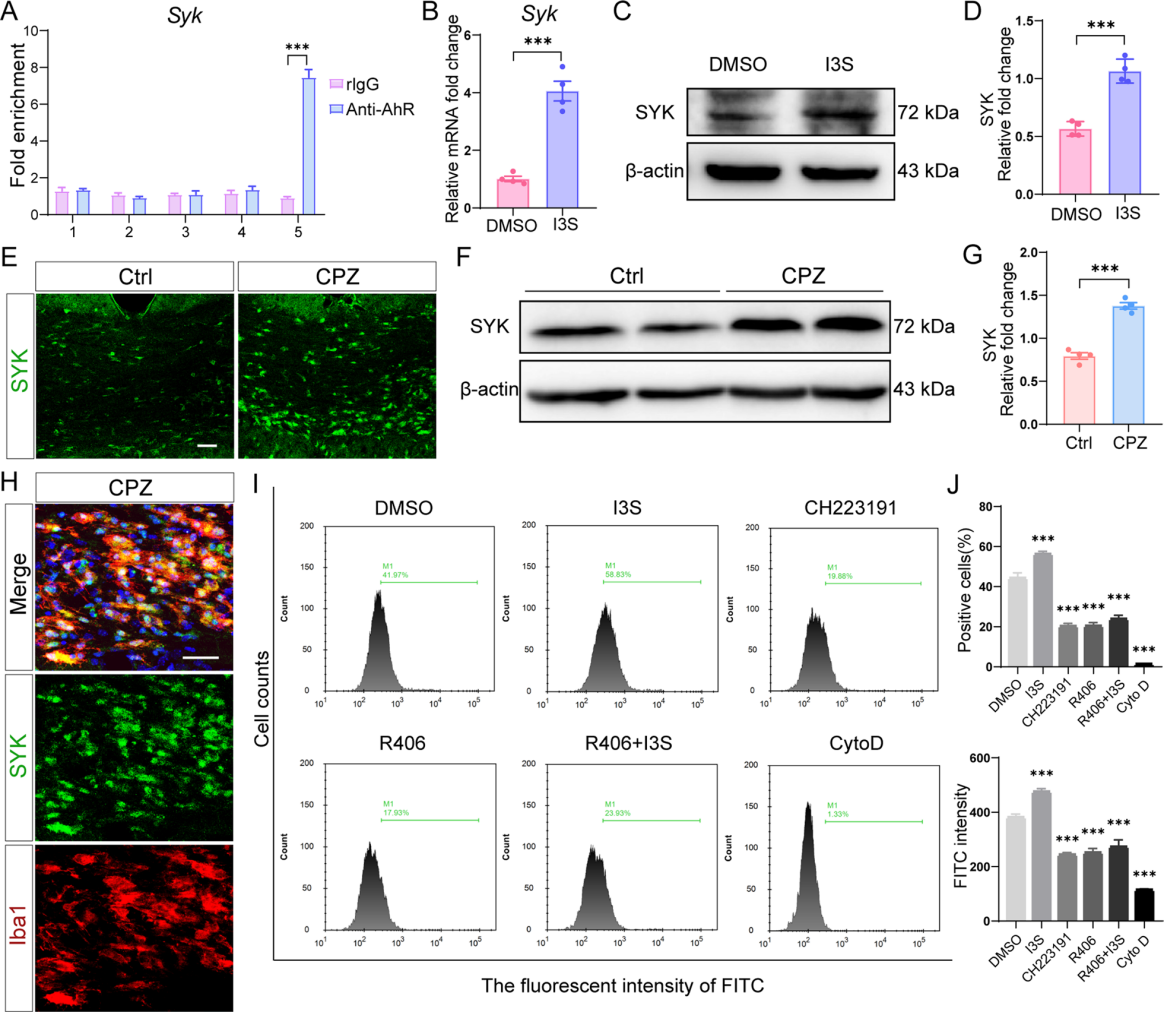
AhR-SYK signaling mediates phagocytic uptake of microglia. **A** ChIP-qPCR analysis was performed in WT microglia to detect the binding of promoter sequences for AhR to SYK (n = 3 biological replicates). The precipitated chromosome segment was PCR-amplified with the use of 5 pairs of specific primers in the SYK promoter. The rlgG is normal rabbit IgG, the homotype control antibody of AhR antibody. Data are shown as mean ± SEM and analyzed by unpaired two-tailed t-test. **B** mRNA expression of *Syk* in primary microglia cultured in vitro and treated with DMSO or AhR agonist I3S (n = 4 biological replicates). Data are shown as mean ± SEM and analyzed by unpaired two-tailed t-test. **C** Western blot analysis of SYK protein level in primary microglia treated with DMSO or AhR agonist I3S. 30 μg protein was loaded per well. **D** The quantification of SYK protein expression in **C** (n = 4 biological replicates). Data are shown as mean ± SEM and analyzed by unpaired two-tailed t-test. **E** Representative confocal immunofluorescent images displaying SYK expression in the corpus callosum of control mice and cuprizone-treated mice (after a 5-week cuprizone administration). Scale bar: 40 μm. **F** Representative western blot analysis of SYK protein level in the corpus callosum of control mice and cuprizone-treated mice (in the context of the end of week 5 in the cuprizone model). 30 μg protein was loaded per well. **G** The quantification of SYK protein expression in **F** (n = 4 mice per group). Data are shown as mean ± SEM and analyzed by unpaired two-tailed t-test. **H** Representative confocal immunofluorescent images displaying co-staining of SYK (green) and Iba1 (red) in the corpus callosum of mice after 5 weeks of cuprizone treatment. Scale bar: 40 μm. **I** Flow cytometry analysis of the phagocytosed pHrodo green bioparticles by HMC3 cells treated as indicated (n = 3 biological replicates). **J** The quantification of positive cells engulfing pHrodo green bioparticles and FITC fluorescent intensity in each group (n = 3 biological replicates). Data are shown as mean ± SEM and analyzed by one-way ANOVA with Tukey's multiple comparisons test. ***p < 0.001

After discovering that microglial AhR deletion impaired phagocytic capacity of microglia, we wanted to see whether AhR, as a transcription factor, directly modulates the expression of phagocytic genes and thus affected phagocytic function. Our RNA-seq data revealed that the phagocytosis related genes *Itgam*, *Calr*, *Pdia6*, *Rab27a*, *Sh3bp1*, and *Syk* are not sufficiently induced in AhR conditional knockout mice following cuprizone treatment (Fig. 5I and J) We then performed chromatin immunoprecipitation (ChIP) analysis on these differential genes of phagocytosis signaling pathway and discovered that AhR was recruited to SYK promoter and calreticulin (calr) promoter in WT primary microglia in culture (Fig. 7A and Additional file 6A). Treatment with AhR agonist I3S upregulated SYK expression at both the transcriptional and translational levels in cultured primary microglia (Fig. 7B–D), while I3S had no effect on calr expression (Additional file 6B and C), suggesting that AhR directly regulates SYK expression.

RNA-seq showed that SYK expression was increased after cuprizone administration, while microglial AhR deficiency significantly downregulated SYK expression (Fig. 5J). As assessed by immunofluorescence staining and western blot, the expression of SYK in the corpus callosum was significantly upregulated in the context of the end of week 5 in the cuprizone model (Fig. 7E–G). Moreover, double immunofluorescence staining showed that in the corpus callosum of cuprizone-treated mice, SYK was mainly colocalized with microglia (Fig. 7H).

SYK is essential for phagocytic function [32, 33]. Next, we investigated whether AhR influences phagocytosis by regulating the phagocytosis-related gene SYK. We expanded our investigation to include human cells. We explored the phagocytic uptake of human microglia-HMC3 cells by flow cytometry. The results showed that after administration of AhR agonist I3S, the percentage of cells engulfing pHrodo green bioparticles was increased and the fluorescence intensity of FITC was upregulated (Fig. 7I and J), indicating the enhanced phagocytic capacity of HMC3 cells. The AhR antagonist CH223191 significantly reduced the phagocytic ability of HMC3 cells (Fig. 7I and J). In line with AhR antagonist, treatment with SYK antagonist R406 also resulted in an apparent defect in phagocytosis (Fig. 7I and J). However, AhR agonist administration failed to reverse the impaired phagocytic function of HMC3 cells caused by SYK antagonist (Fig. 7I and J). These findings indicated that AhR modulates SYK expression and thus affects phagocytic uptake of cells, confirming the molecular mechanism of AhR transcriptional regulation on phagocytic genes.

### Microglial AhR deficiency exacerbates CNS inflammation

The 214 differential genes (Fig. 5E), which were uniquely upregulated in CX3CR1-AhR^−/−^ mice after demyelination, were involved in inflammatory signaling pathways such as JAK-STAT signaling pathway and NOD-like receptor signaling pathway, as well as apoptosis, cytokine-cytokine receptor interaction, TNF signaling pathway and inflammatory response (Additional file 7A and B). In other words, inflammatory pathways were activated in AhR conditional knockout mice.

Hematoxylin–eosin (HE) staining demonstrated hypercellularity and showed a large number of inflammatory cell accumulation in the corpus callosum after cuprizone administration (Additional file 7C), while the number of inflammatory cell accumulation in AhR conditional knockout mice was significantly higher than that in littermate control mice (Additional file 7C). Moreover, we detected the expression of pro-inflammatory factors in the corpus callosum and found that the transcriptional level of *Nos2, Il1b, Il6* and *Ccl2* were significantly increased in AhR conditional knockout mice of cuprizone model (Additional file 7D–G). Overall, deletion of microglial AhR worsens the CNS inflammation response during demyelination.

## Discussion

Here we report that microglial AhR modulates the expression of SYK to enhance the phagocytosis of myelin debris by microglia, thereby further affecting myelin regeneration. Our study highlights the role of AhR in microglial function and CNS remyelination. It is well known that microglia are critical in remyelination. Clinical trials have confirmed that certain drugs trialed for remyelination have beneficial effects on microglia responses [34–36], raising the possibility of directly targeting microglia or specific molecules in microglia to promote remyelination. However, the heterogeneity of microglia adds challenges to the development of drugs targeting microglia. Microglia populations are phenotypically and functionally divided into distinct subsets [37, 38]. We have also observed that the morphology of microglia in the corpus callosum of our concern is significantly different from that in other sites. The heterogeneity of microglia in the transcriptome and protein expression is likely to affect their ability to modulate remyelination. In addition, microglia go through changes in gene expression and function during the remyelination process [5, 39, 40]. Comprehending the different functional properties of microglia subsets and effector molecules may be conducive to developing effective therapies.

Our study demonstrates that microglial AhR regulates the engulfment of myelin debris by microglia. The effect of AhR on microglial phagocytic function is rarely reported. The clearance of myelin debris by microglia is manipulated by a complex network involving a variety of enzymes and receptors. The activation of transient receptor potential vanilloid 1 (TRPV1) promotes the expression of the scavenger receptor CD36 and potentiates myelin debris clearance [41]. Mertk is required for competent microglial phagocytosis and it has been indicated that Mertk knockout mice exhibit impaired clearance of myelin debris in lesions compared to WT mice at 4-week time point of demyelination model. Nevertheless, vast majority of myelin debris is phagocytosed in Mertk knockout mice at 4 + 3-week of demyelination model, suggesting a compensatory mechanism that does not require Mertk to clear myelin debris is engaged [42]. Additionally, Cx3cr1 knockout inhibits the phagocytosis of myelin debris by microglia, resulting in aberrant remyelination. Whereas subsequent remyelination still occurs, implying the existence of a compensatory mechanism not entailing Cx3cr1 [43]. We have not extended the time course to explore whether the uptake of myelin debris by microglia is restored in CNS of AhR conditional knockout mice. There may also be compensatory mechanisms which deserve further exploration.

In our study, we report that AhR modulates SYK expression and thus affects the phagocytic function of microglia. SYK is a key player in phagocytosis which mediates the downstream signaling of many phagocytic receptors such as Fcγ receptor, Dectin-1 and complement receptor [44, 45]. Our in vitro experiments reveal that AhR activation enhances the phagocytic ability of human microglia-HMC3 cells, however, this effect is abolished by a SYK antagonist. SYK also has a central role in the autoimmune diseases [46, 47]. Our research confirms that SYK is involved during cuprizone-induced demyelination. Recent studies have demonstrated that SYK deletion leads to exacerbated Aβ plaque deposition and aggravated neuropathology in an AD mouse model [48, 49]. SYK critically regulates microglial phagocytic clearance of neurotoxic material in models of AD and MS [48]. These findings corroborate our study. Moreover, our study has proved that AhR, the upstream of SYK, modulates microglial phagocytosis by SYK.

In addition to the phagocytic uptake of myelin debris, the removal of myelin debris by microglia after demyelination involves subsequent degradation process [5]. Our RNA-seq results reveal that microglial AhR deficiency prevents the increased expression of lysosome pathway-related genes, as well as the increase in the expression of genes related to lipid transport and lipid catabolic process signaling pathways. Moreover, we identify that in cuprizone model, the morphology of microglia changes significantly in the corpus callosum of AhR conditional knockout mice compared to littermate control mice. We suspect that this may be the manifestation of damaged lysosomal degradation function in microglia. Hereafter we intend to focus on whether AhR affects the subsequent degradation of myelin debris by microglia.

Consistent with previous reports on the role of AhR in inflammation [16, 26], our study finds that AhR conditional knockout promotes the expression of pro-inflammatory factors in the corpus callosum and exacerbates the inflammatory response during demyelination. In the colitis model, AhR knockout mice show more severe symptoms and higher expression of proinflammatory cytokines than WT mice [50]. Further, AhR also plays an essential role in suppressing inflammation in the EAE model as mentioned earlier [20].

Our study detects that exposure to cuprizone induces increased AhR expression and transcriptional activity in the corpus callosum. The underlying mechanism of AhR activation in cuprizone model is an open question. The ligand binding site of AhR is physiologically flexible and a number of small molecules are capable of serving as ligands to activate AhR [51]. There are many endogenous AhR ligands, and most of them are derived from tryptophan metabolites such as kynurenine, indoleacetic acid, 6-formylindolo[3,2-b]carbazole (FICZ), as well as I3S which is used in our study. In inflammatory states, indoleamine 2,3-dioxygenase mediates the metabolism of tryptophan to kynurenine, which subsequently activates AhR [15, 16]. A recent study has shown that high level of IL-2 induces the conversion of tryptophan to 5-hydroxytryptophan (5-HTP), and 5-HTP activates AhR, thereby causing T cells dysfunctional in the tumor microenvironment [52]. Furthermore, IFN-I signaling in astrocytes activates AhR and thus reduces CNS inflammatory response and EAE disease scores [20]. In conclusion, diet and metabolism provide a variety of AhR agonists, and AhR activation also involves multiple upstream signaling pathways. Developing drugs targeting these agonists and associated pathways may turn into novel treatments for inflammatory and neurodegenerative diseases. Taken together, our study provides a promising strategy for targeting AhR in microglia for the therapies of CNS demyelinating diseases.

## Methods

### Animals and tamoxifen administration

Wild-type mice were provided by the Shanghai Experimental Animal Center of the Chinese Academy of Sciences. AhR^fl/fl^ mice (JAX stock 006203) were obtained from The Jackson Laboratory. CX3CR1^creERT2^ mice (JAX stock 021160) were gifted by Prof. Yuqiu Zhang, Fudan University, China. All mice used were male and were of the C57BL/6 genetic background. Mice were kept under standard conditions at 23° ± 3 °C and a 12-h light–dark cycle. All animal experiments were performed according to the guidelines of the Animal Ethics Committee of Shanghai Medical College, Fudan University, China.

Tamoxifen (Sigma-Aldrich, USA) was dissolved in corn oil (Absin, China) at 20 mg/mL. To induce Cre-mediated recombination, 4-week-old mice received intraperitoneal injections of tamoxifen (200 mg/kg) every other day for three times. Cuprizone treatment were performed 3 weeks after tamoxifen administration.

### Cuprizone treatment

Six-week-old male mice were fed a diet containing 0.3% cuprizone (Sigma-Aldrich, USA) mixed in standard powdered rodent chow to induce oligodendrocyte death and demyelination. Food was replaced every other day. After 5 weeks of cuprizone treatment, mice were fed a normal standard diet for one week. Mice were sacrificed at 5 and 6 weeks after cuprizone administration.

### CH223191 and I3S administration

Mice in antagonist group were intraperitoneally injected with CH223191 daily at a dose of 10 mg/kg for 3 weeks. Mice in agonist group were intraperitoneally injected with I3S daily at a dose of 5 mg/kg for 3 weeks.

### Beam walking test

The beam walking apparatus consisted of a wooden bar (width of 1 cm and length of 100 cm) placed 60 cm above the ground and a non-transparent box. Mice were placed on the other end of the beam and were trained to walk through the beam to the box (cut-off time 20 s). Then recorded the traversing time in each trial and wiped the apparatus with 75% ethanol after each trial to avoid smell cues.

### Rotarod test

During each trial, mice were placed on the rotating rod and the rate of the rod began accelerating gradually from 4 to 40 rpm within 5 min. Latency to fall from the rotarod was automatically recorded and the average was calculated from three trials with an interval of 30 min.

### qRT-PCR

RNA from the mouse corpus callosum tissues and cells was extracted using the Qiagen RNeasy kit (74106, Qiagen, Germany) according to the manufacturer protocol and the concentration of RNA was measured by nanodrop (Thermo Fisher, USA). After that, cDNA was prepared using the PrimeScript RT Master Mix Reverse Transcription Kit (RR036, Takara, Japan) according to the manufacturer protocol. Gene expression was determined by qRT-PCR on 7300Plus Real-Time PCR System (Applied Biosystems, USA) using TB Green Premix Ex Taq (RR420, Takara, Japan). Relative gene expression was normalized to the expression of housekeeping gene GAPDH. Primers used are listed below.

Mouse: *Gapdh* (Forward 5′GACCCCTTCATTGACCTCAAC3′, Reverse 5′GATGACCTTGCCCACAGCCTT3′),

*Ahr* (Forward 5′AGGACCAGTGTAGAGCACAAA3′, Reverse 5′AAGCATAGAAGACCAAGGCA3′),

*Arnt* (Forward 5′ATCGGTCTAGTTCCAGTGAGC3′, Reverse 5’TGTAGGTGTTGCTTTGGTCTC3′),

*Cyp1a1* (Forward 5′TCTTCAGGCTTAGACTGTCCA3′, Reverse 5’CAGGATCTGTGTTTCTGGCTA3′),

*Syk* (Forward 5′GGCTCACAACAGGAAGGCACAC3′, Reverse 5′TGGGAGTGGTAATGGCAGAGGTC′),

*Nos2* (Forward 5′TCCAGAATCCCTGGACAAGCTGC3′, Reverse 5′TGCAAGTGAAATCCGATGTGGCCT3′),

*Il-1b* (Forward 5′TACATCAGCACCTCACAAGC3′, Reverse 5′AGAAACAGTCCAGCCCATACT3′),

*Il-6* (Forward 5′CTTCTTGGGACTGATGCTGGTGAC3′, Reverse 5′TCTGTTGGGAGTGGTATCCTCTGTG3′),

*Ccl2* (Forward 5′CACTCACCTGCTGCTACTCATTCAC3′, Reverse 5′CTTCTTTGGGACACCTGCTGCTG3′).

### Western blotting

The entire corpus callosum tissue was meticulously detached from brain on the ice with elbow ophthalmic forceps and ophthalmic scissors, followed by ultrasonic homogenization in lysis buffer (Beyotime Biotechnology, China) containing PMSF. Protein lysates obtained were centrifuged at 12,000 rpm for 15 min and supernatants were quantified using BCA assay kit (Thermo Fisher, USA). Protein samples were run with 10% SDS-PAGE gels and proteins were transferred onto PVDF membranes (Millipore, USA). The PVDF membranes were incubated with primary antibodies including mouse anti-AhR (1:1000, MA1-513, Thermo Fisher, USA), rabbit anti-MBP (1:1000, ab40390, Abcam, UK), rabbit anti-β-actin (1: 9000, 60004, Proteintech, USA), rabbit anti-Histone H3 (1:2000, 4499, CST, USA), rabbit anti-SYK (1:1000, 2717, CST, USA) and rabbit anti-Calr (1:1000, 12238, CST, USA). Using Immobilon Western HRP Substrate (Millipore, USA) to detect proteins after incubating the PVDF membranes with anti-rabbit HRP-linked secondary antibody for 2 h at RT. HRP-conjugated blots were detected by using ImageQuant LAS4000 mini-image analyzer (GE Healthcare, UK).

### Immunofluorescence

Mice were perfused with 1 × PBS followed by 4% PFA and then mouse brains were dissected. The frozen brain sections were prepared after brains were fixed and dehydrated. The frozen sections were washed three times for 5 min in PBS and blocked by 5% goat serum for 2 h, followed by incubation with primary antibodies overnight at 4 °C. Primary antibodies included mouse anti-AhR (1:500, MA1-513, Thermo Fisher, USA), rabbit anti-MBP (1:1000, ab40390, Abcam, UK), rabbit anti-NeuN (1:300, ab177487, Abcam, UK), rabbit anti-Olig2 (1:300, AB9610, Millipore, USA), rabbit anti-GFAP(1:600, 80788, CST, USA), rabbit anti-Iba1 (1:500, 019-19741, Wako, Japan), mouse anti-CC1 (1:500, OP80, Millipore, USA), rabbit anti-dMBP (1:400, AB5864, Millipore, USA) and rabbit anti-SYK (1:500, 2717, CST, USA). The frozen sections were washed again after primary antibody incubation and incubated with Alexa Fluor 488-labeled or 594-labeled secondary antibodies (1:1000, Invitrogen, USA) for 2 h. Sections were washed three times and mounted by Fluoromount-G with DAPI.

### Electron microscopy

Mice were perfused with cold PBS followed by fixative containing 2.5% glutaraldehyde and 4% PFA. Brains were removed and the corpus callosum tissues were sliced into 1 mm sagittal slices. After fixed in fixative at least 24 h, sagittal slices were dehydrated in ethanol and embedded in embedding medium. Ultrathin sections were cut on UC7 ultramicrotome (Leica, Germany) and representative pictures were taken under FEI TECNAI G2 20 TWIN electron microscope. The number of myelinated axons in ultrathin sections was analyzed for quantification.

### RNA sequencing and data processing

Total RNA was extracted from mouse corpus callosum using the Qiagen RNeasy kit (74106, Qiagen, Germany) according to the manufacturer protocol. Total RNA was quantified using nanodrop (Thermo Fisher, USA) before sending to ANOROAD (Beijing, China) for preparing Libraries and sequencing. Concentration and integrity of RNA were determined by Agilent 2100 RNA nano 6000 assay kit (Agilent Technologies, USA). A total amount of 1–3 μg RNA per sample was used to prepare libraries using VAHTS Universal V6 RNA-seq Library Prep Kit for Illumina (NR604-01/02) according to the manufacturer’s protocol and index codes were added to attribute sequences to each sample. RNA concentration of library was measured using Qubit RNA Assay Kit. The clustering of the index-coded samples was performed using HiSeq PE Cluster Kit v4-cBot-HS (Illumina) following the manufacturer’s recommendations. After cluster generation, the libraries were sequenced on Novaseq 6000 S4 platform, using NovaSeq 6000 S4 Reagent kit V1.5. After sequencing and preparing Libraries, FPKM values obtained from raw files were used for gene expression analysis. Removing genes with 0 read counts and low expression and normalizing the data. Using DESeq2 for differential analysis. Thresholds for differential expression genes are FDR < 0.05 and fold change > 1.5. GO enrichment analyses and KEGG pathway analysis were generated using Omicsbean (http://www.omicsbean.cn/).

### Oil red O staining

Frozen sections were prepared as described earlier and were gently rinsed in 60% isopropanol for 60 s. Subsequently, the sections were stained with Oil Red O working solution (Sigma-Aldrich, USA) freshly prepared at 60 °C for 20 min. After that the sections were incubated with hematoxylin to stain the nucleus.

### Myelin preparation

Myelin was isolated from 12-week-old C57BL/6J mouse brains. Specifically, brains were homogenized in 0.32 M sucrose solution and then added 0.85 M sucrose solution slowly to the liquid surface followed by centrifugation at 75,000 × g for 30 min. The interphase was collected, which is roughly purified myelin. Myelin was washed with sterilepure water and centrifuged at 12,000 × g for 15 min. The pellet was collected and resuspended in 0.35 M sucrose solution. After that, 0.85 M sucrose solution was added gently to the liquid surface as described earlier. After centrifuged at 75,000 × g for 30 min, purified myelin was washed with sterile pure water and centrifuged at 12,000 × g for 15 min. The pellet was resuspended in PBS and stored at − 80 °C.

### Primary microglia culture and HMC3 cell culture

Primary microglia were isolated from mixed glial of P0-2 neonatal mice. Specifically, mixed glial were resuspended in DMEM medium (Gibco, USA) supplemented with l-glutamine (Sigma-Aldrich, USA) and 10% Fetal bovine serum (FBS, Gibco, USA) and plated on T75 flasks (Corning, USA) for 2 weeks. Primary microglia were collected after 2 weeks by shaking the flasks with an orbital shaker at 200 r.p.m for 2 h. One day after plated on plates, microglia from AhR^fl/fl^ and AhR^fl/fl^ CX3CR1^creERT2^ mice were treated with 0.1 μM 4-OH tamoxifen (Sigma-Aldrich, USA) for 2 days. For phagocytosis assay, isolated microglia were cultured on poly-d-lysine-coated 12-well plates at 5 × 10^5^ cells per ml in DMEM medium with 10% FBS and 20 ng/ml m-CSF (R&D systems, USA). For immunofluorescence assay, microglia were plated at 8 × 10^4^ cells per ml. The Human Microglia Clone 3 (HMC3) cell line was purchased from the Procell. HMC3 cells were maintained in MEM + 10% FBS + 1%P/S (Procell, China) and plated on poly-d-lysine-coated 12-well plates at a density of 5 × 10^5^ cells per ml for western blot and phagocytosis assay. All cells were cultured in 5% CO_2_ at 37 °C.

### Phagocytosis assay

Primary microglia and HMC3 cells were plated into 12-well plates as described above. After 24 h, all cells were treated with myelin debris at 10 μg/mL. PHrodo E. coli bioparticles (P35366, Thermo Fisher, USA) were added to the cells for 2 h to evaluate the phagocytic ability. As negative controls, pre-treatment of Cytochalasin D was carried out for 30 min to prevent phagocytic uptake of cells. For fluorescence staining, cells were washed with PBS to remove non-phagocytosed pHrodo bioparticles followed by fixation with 4% PFA for 30 min. After blocked by 5% goat serum, cells were incubated with primary antibody rabbit anti-Iba1 (1:500, Wako, Japan) overnight at 4 °C. Cells were incubated with 594-labeled secondary antibodies (Invitrogen, USA) for 2 h and then mounted by Fluoromount-G with DAPI for 10 min. For flow cytometry assay, cells were trypsinized and centrifuged at 1000 rpm for 5 min. After that, cells were resuspended in FACS buffer to analyze the fluorescent intensity of FITC on a FACSVerse Flow Cytometer (BD Biosciences, USA).

### Chromatin immunoprecipitation (ChIP)

Preparing approximately 4 X 106 cells per sample for ChIP and using SimpleChIP Plus Enzymatic Chromatin IP Kit (9005, CST, USA) for ChIP sample preparation. Cells were cross-linked with 1% formaldehyde for 20 min. Subsequently, terminating cross-linking by using glycine for 5 min and then adding micrococcal nuclease to samples to obtain 150–900 bp length chromosome segment. Chromatin lysates were sonicated with sonicator to disrupt the nuclear membrane. After that, ChIP samples were incubated with rabbit anti-AhR (1:100, MA1-514, Thermo Fisher, USA) and rabbit anti-Histone H3 (1:50, 4620, CST, USA) and negative control was incubated with normal rabbit IgG at 4 °C overnight. Adding Protein G Magnetic Beads to ChIP samples on a rotter for 2 h at 4 °C. The beads were washed with elution buffer to elute chromatin and eluted chromatin supernatant was heated at 65 °C for 2 h to reverse the cross-linking, followed by DNA purification using spin columns. For ChIP-qPCR, the following primer sequences were used:

*Syk*-1 (Forward 5′ATGGCTCTCAGTGGGGTGTC3’, Reverse 5’GCAGGTCTCAGGGATCAAGGT3′),

*Syk*-2 (Forward 5′GGTTAGCACAGGGTCACAGC3′, Reverse 5’CAGTCATGGTGTCTCCCAGC3’),

*Syk*-3 (Forward 5′TCTGGCGTCACCCAATTCCT3′, Reverse 5′TCTGCTGTTCCTTGCTCACAG3′),

*Syk*-4 (Forward 5′CCAGGCCCCAGTTCTGTGTA3′, Reverse 5′GTACAAAGGCCACCAGAGCC3′),

*Syk*-5 (Forward 5′GCATGTCAAGGACTGCAGGA3′, Reverse 5′GCCAACACCATGGCATGAGA3’),

*Calr*-1 (Forward 5′GTAATGGGATCCAATGCCCTCTTCT3′, Reverse 5’TTCTGAATGACCCCAGTCCTATTCCC3’),

*Calr*-2 (Forward 5′TTGATCCTTCATGTGTGTGTGCTGG3′, Reverse 5′ATGGGAGGTGTTTCCAAACTGAG3′),

*Calr*-3 (Forward 5′AGTCTTGTTTCAGGGTTCCCAATTCC3′, Reverse 5′TGTGTGACATGATACAAGTCATTTCGCC3′),

*Calr*-4 (Forward 5′TTGGCTTCTGACATTTCAGGGAGG3′, Reverse 5′ACTATGGGCCAATGAGGGTCGA3′),

*Calr*-5 (Forward 5′CTGAGTGGGCTAGCGGC3′, Reverse 5′ATTACACACGACGAGGCAGC3′).

### Statistical analysis

All data were analyzed by GraphPad 8.0 software. Data were compared using unpaired two-tailed t-test for two groups. As for multiple group comparison, one-way ANOVA or two-way ANOVA was performed as labeled separately in the figure legends. p values < 0.05 were considered statistically significant and data were expressed as mean values ± SEM.

## Supplementary Information

**Additional file 1.** Further activation of AhR enhances remyelination in cuprizone model. (A) Time course of agonist I3S administration in cuprizone model. (B) Body weight of control mice and cuprizone-treated mice with or without I3S administration (n = 7 mice per group). Data are shown as mean ± SEM and analyzed by two-way ANOVA with Tukey's multiple comparisons test. (C) Beam walking test and rotarod test evaluating motor coordinative function were performed in 4 experimental groups (n = 7 mice per group). Data are shown as mean ± SEM and analyzed by two-way ANOVA with Tukey's multiple comparisons test. (D) Representative confocal immunofluorescent images displaying MBP expression in the corpus callosum of control mice and cuprizone-treated mice with or without I3S treatment. Scale bar: 40 μm. (E) The quantification of MBP fluorescence intensity in the corpus callosum in (D) (n = 4 mice per group). Data are shown as mean ± SEM and analyzed by two-way ANOVA with Tukey's multiple comparisons test. (F) Representative confocal immunofluorescent images of CC1 staining in the corpus callosum of control mice and cuprizone-treated mice with or without I3S administration. Scale bar: 40 μm. (G) The quantification of CC1 + mature oligodendrocytes in the corpus callosum in (F) (n = 4 mice per group). Data are shown as mean ± SEM and analyzed by two-way ANOVA with Tukey’s multiple comparisons test. **p < 0.01, ***p < 0.001, ns (not significant). The results of Additional file 1 are acquired at the end of week 6 of the cuprizone model.

**Additional file 2.** Validation of efficient deletion of AhR in microglia. (A) Representative confocal immunofluorescent images of AhR (green) and Iba1 (red) in the corpus callosum of CX3CR1-AhR^−/−^ mice in cuprizone model. Scale bar: 40 μm. (B) mRNA expression of *Cyp1a1* in the corpus callosum of AhR^fl/fl^ mice and CX3CR1-AhR^−/−^ mice was evaluated by qRT-PCR (n = 4 mice per group). Data are shown as mean ± SEM and analyzed by unpaired two-tailed t-test. ∗ p < 0.05. The results of Additional file 2 are acquired in the context of the end of week 5 in the cuprizone model.

**Additional file 3.** Microglial AhR deficiency has no effect on demyelination. (A) Representative confocal immunofluorescent images of MBP in the corpus callosum of AhR^fl/fl^ mice and CX3CR1-AhR^−/−^ mice after a 5-week cuprizone administration. Scale bar: 40 μm. (B) The quantification of MBP fluorescence intensity in the corpus callosum in (A) (n = 4 mice per group). Data are shown as mean ± SEM and analyzed by two-way ANOVA with Tukey's multiple comparisons test. ***p < 0.001, ns (not significant).

**Additional file 4.** Microglial AhR deficiency alters microglial morphological changes. (A) Representative confocal immunofluorescent images of Iba1 in the corpus callosum of AhR^fl/fl^ mice and CX3CR1-AhR^−/−^ mice after a 5-week cuprizone administration. Scale bar: 40 μm. Representative skeletonized images of microglia in the 4 experimental groups corresponding to the white box. (B) The quantification of Iba1 positive staining in the corpus callosum in (A) (n = 4 mice per group). Data are shown as mean ± SEM and analyzed by two-way ANOVA with Tukey’s multiple comparisons test. (C and D) The quantification of microglial process endpoints and process length per cell in 4 experimental groups (n = 4 mice per group). Data are shown as mean ± SEM and analyzed by two-way ANOVA with Tukey's multiple comparisons test. *p < 0.05, **p < 0.01, ***p < 0.001.

**Additional file 5.** AhR has no effect on microglia viability and migration in vitro. (A) Viability of primary microglia cultured in vitro and treated with DMSO or AhR agonist I3S was analyzed by CellTiter-Glo assay (n = 3 biological replicates). Data are shown as mean ± SEM and analyzed by unpaired two-tailed t-test. (B) Representative images displaying the wound scratch assay of primary microglia treated with DMSO or AhR agonist I3S. Scale bar: 200 μm. (C) Representative images of transwell assay. Primary microglia migrating to the lower compartment were stained with DAPI. Scale bar: 100 μm. (D) The quantification of migrated primary microglia in (D) (n = 3 biological replicates). Data are shown as mean ± SEM and analyzed by unpaired two-tailed t-test. ns (not significant).

**Additional file 6.** AhR agonist I3S has no effect on calr expression. (A) ChIP-qPCR analysis was performed in WT microglia to detect the binding of promoter sequences for AhR to Calr (n = 3 biological replicates). The precipitated chromosome segment was PCR-amplified with the use of 5 pairs of specific primers in the Calr promoter. The rlgG is normal rabbit IgG, the homotype control antibody of AhR antibody. Data are shown as mean ± SEM and analyzed by unpaired two-tailed t-test. (B) Western blot analysis of calr protein level in primary microglia treated with DMSO or AhR agonist I3S. 30 μg protein was loaded per well. (C) The quantification of calr protein expression in (I) (n = 3 biological replicates). Data are shown as mean ± SEM and analyzed by unpaired two-tailed t-test. ***p < 0.001, ns (not significant).

**Additional file 7. **AhR deficiency in microglia aggravates CNS inflammation. (A) KEGG pathway analysis of 214 upregulated genes unique to CX3CR1-AhR^−/−^ mice after cuprizone administration. (B) GO term enrichment analysis of 214 upregulated genes unique to CX3CR1-AhR^−/−^ mice after cuprizone administration. (C) Representative HE stained images in the corpus callosum of AhR^fl/fl^ mice and CX3CR1-AhR^−/−^ mice in the context of the end of week 6 in the cuprizone model. Scale bar: 50 μm. (D-G) mRNA expressions of *Nos2*, *Il1b*, *Il6* and *Ccl2* in the corpus callosum of AhR^fl/fl^ mice and CX3CR1-AhR^−/−^ mice at the end of week 6 of cuprizone model were evaluated by qRT-PCR (n = 4 mice per group). Data are shown as mean ± SEM and analyzed by two-way ANOVA with Tukey's multiple comparisons test. *p < 0.05, **p < 0.01, ***p < 0.001.

## Data Availability

The RNA-seq data are accessible through Gene Expression Omnibus GSE210147 (https://www.ncbi.nlm.nih.gov/geo/query/acc.cgi?acc=GSE210147).

## Abbreviations

MS: Multiple sclerosis
CNS: Central nervous system
CPZ: Cuprizone
AhR: Aryl hydrocarbon receptor
SYK: Spleen tyrosine kinase
AD: Alzheimer’s disease
PD: Parkinson’s disease
bHLH: Basic helix-loop-helix
EAE: Experimental autoimmune encephalitis
MBP: Myelin basic protein
LFB: Luxol Fast Blue
I3S: Indoxyl-3-sulfate
TEM: Transmission electron microscopy
WT: Wild-type
dMBP: Degraded MBP
ChIP: Chromatin immunoprecipitation
Calr: Calreticulin
HE: Hematoxylin–eosin
TRPV1: Transient receptor potential vanilloid 1
FICZ: 6-Formylindolo[3,2-b]carbazole
5-HTP: 5-Hydroxytryptophan
