# Supplementary figures and images for: Microglial aryl hydrocarbon receptor enhances phagocytic function via SYK and promotes remyelination in the cuprizone mouse model of demyelination
Source: J Neuroinflammation. 2023 Mar 25;20:83. doi: 10.1186/s12974-023-02764-3 (PMC10040134; doi:10.1186/s12974-023-02764-3)

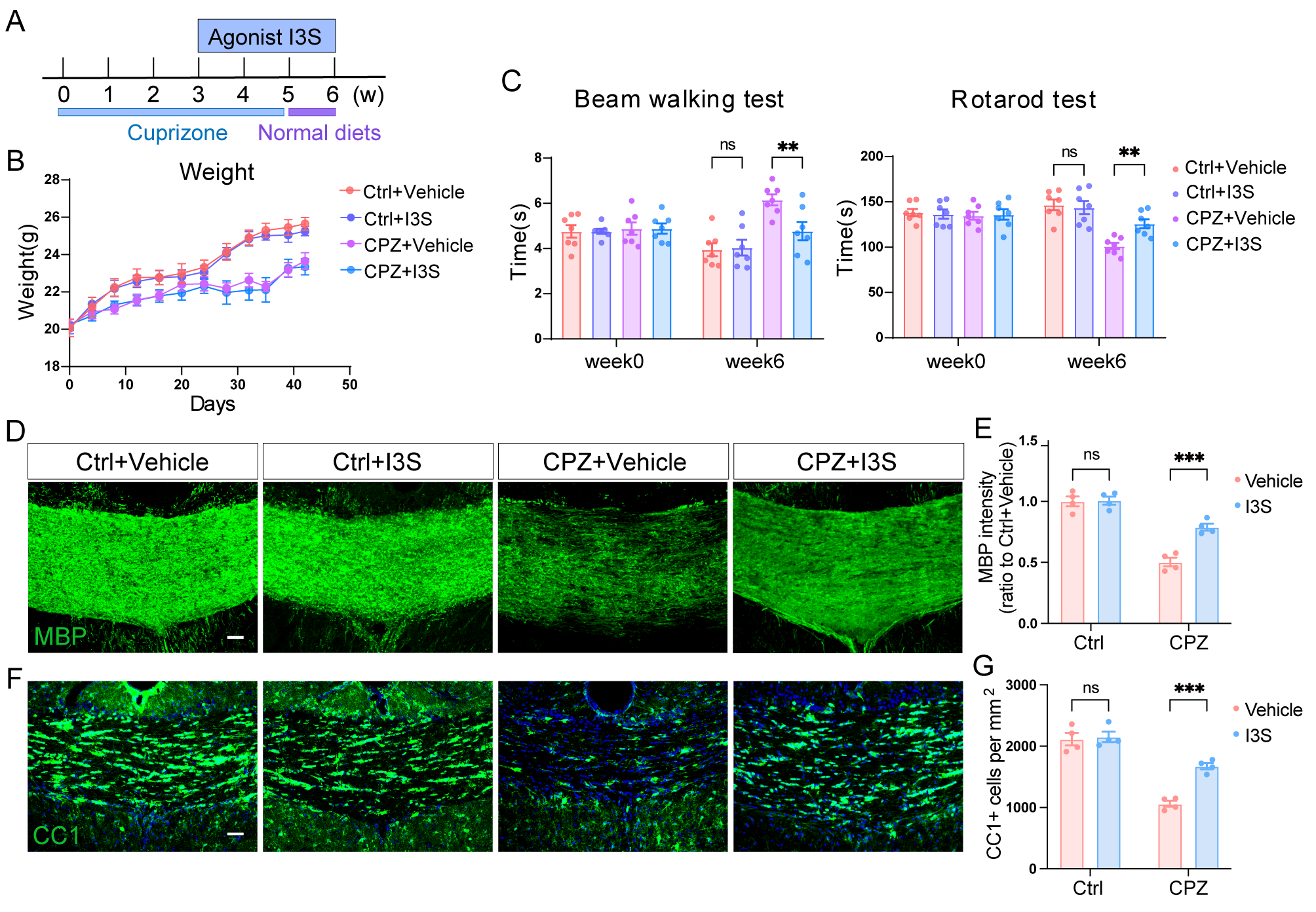

Supplement: Supplementary file 1 — Additional file 1. Further activation of AhR enhances remyelination in cuprizone model. (A) Time course of agonist I3S administration in cuprizone model. (B) Body weight of control mice and cuprizone-treated mice with or without I3S administration (n = 7 mice per group). Data are shown as mean ± SEM and analyzed by two-way ANOVA with Tukey's multiple comparisons test. (C) Beam walking test and rotarod test evaluating motor coordinative function were performed in 4 experimental groups (n = 7 mice per group). Data are shown as mean ± SEM and analyzed by two-way ANOVA with Tukey's multiple comparisons test. (D) Representative confocal immunofluorescent images displaying MBP expression in the corpus callosum of control mice and cuprizone-treated mice with or without I3S treatment. Scale bar: 40 μm. (E) The quantification of MBP fluorescence intensity in the corpus callosum in (D) (n = 4 mice per group). Data are shown as mean ± SEM and analyzed by two-way ANOVA with Tukey's multiple comparisons test. (F) Representative confocal immunofluorescent images of CC1 staining in the corpus callosum of control mice and cuprizone-treated mice with or without I3S administration. Scale bar: 40 μm. (G) The quantification of CC1 + mature oligodendrocytes in the corpus callosum in (F) (n = 4 mice per group). Data are shown as mean ± SEM and analyzed by two-way ANOVA with Tukey’s multiple comparisons test. **p < 0.01, ***p < 0.001, ns (not significant). The results of Additional file 1 are acquired at the end of week 6 of the cuprizone model. [file 12974_2023_2764_MOESM1_ESM.tif]

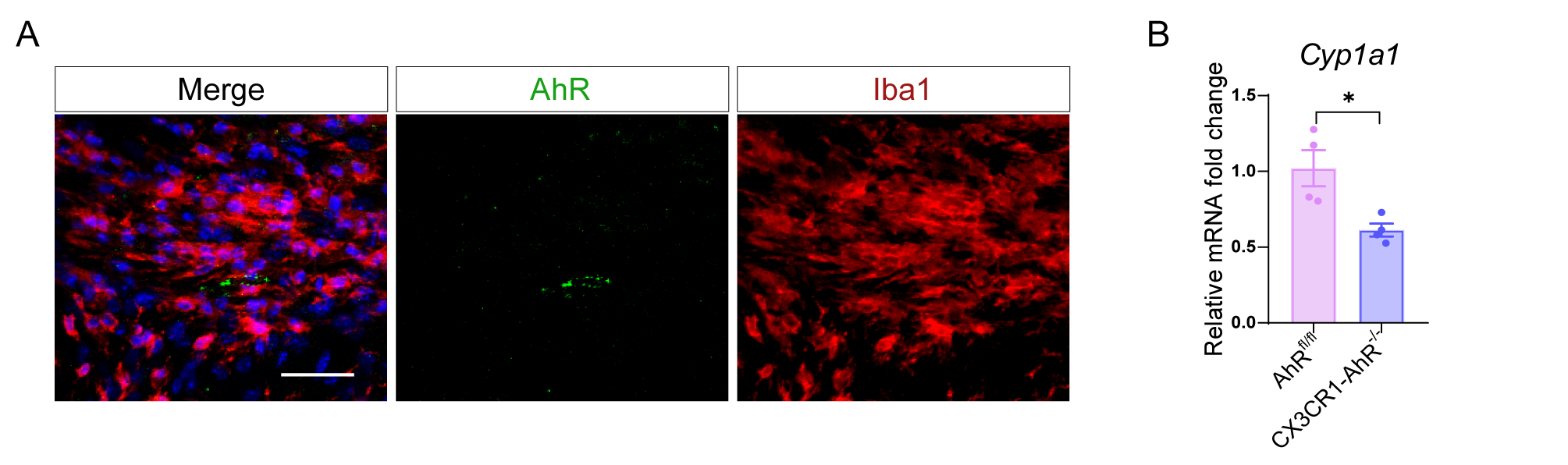

Supplement: Supplementary file 2 — Additional file 2. Validation of efficient deletion of AhR in microglia. (A) Representative confocal immunofluorescent images of AhR (green) and Iba1 (red) in the corpus callosum of CX3CR1-AhR−/− mice in cuprizone model. Scale bar: 40 μm. (B) mRNA expression of Cyp1a1 in the corpus callosum of AhRfl/fl mice and CX3CR1-AhR−/− mice was evaluated by qRT-PCR (n = 4 mice per group). Data are shown as mean ± SEM and analyzed by unpaired two-tailed t-test. ∗ p < 0.05. The results of Additional file 2 are acquired in the context of the end of week 5 in the cuprizone model. [file 12974_2023_2764_MOESM2_ESM.tif]

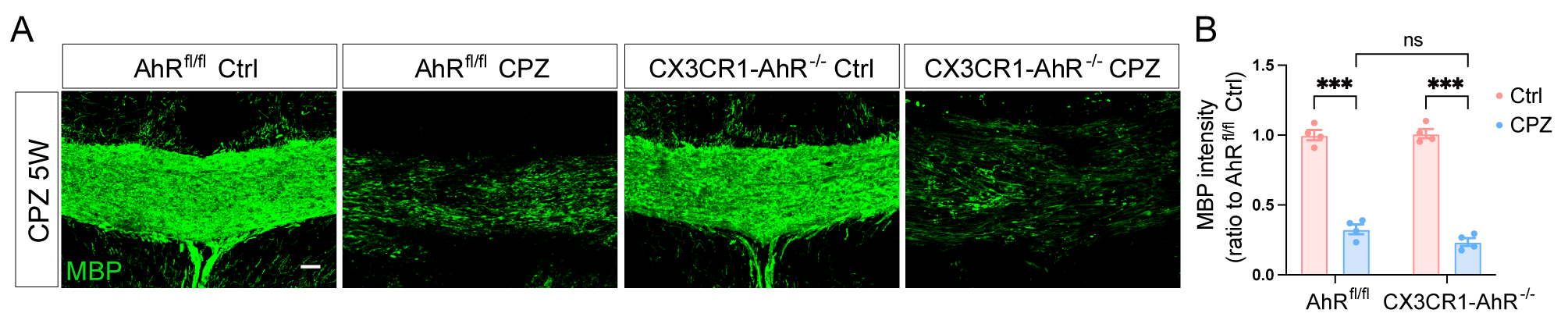

Supplement: Supplementary file 3 — Additional file 3. Microglial AhR deficiency has no effect on demyelination. (A) Representative confocal immunofluorescent images of MBP in the corpus callosum of AhRfl/fl mice and CX3CR1-AhR−/− mice after a 5-week cuprizone administration. Scale bar: 40 μm. (B) The quantification of MBP fluorescence intensity in the corpus callosum in (A) (n = 4 mice per group). Data are shown as mean ± SEM and analyzed by two-way ANOVA with Tukey's multiple comparisons test. ***p < 0.001, ns (not significant). [file 12974_2023_2764_MOESM3_ESM.tif]

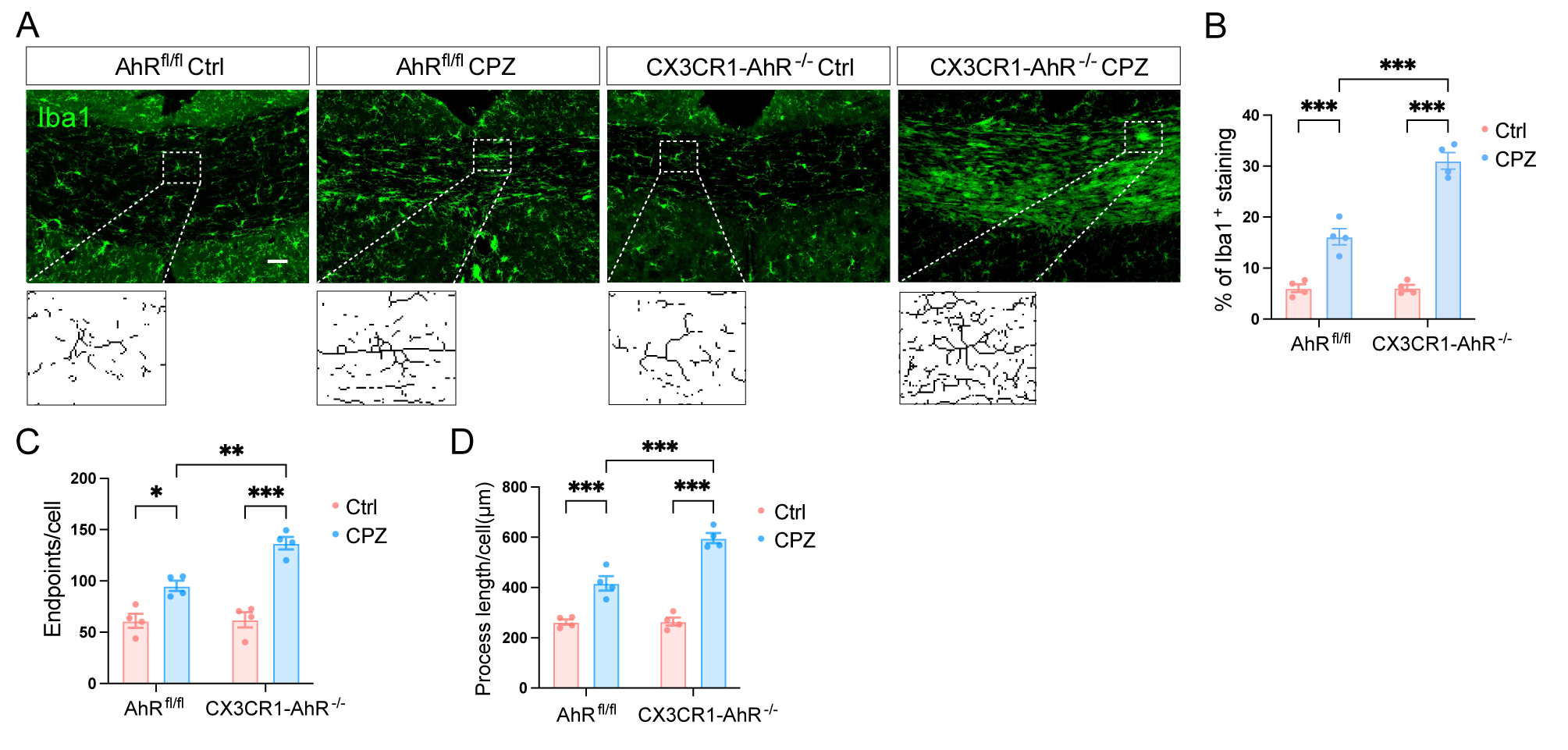

Supplement: Supplementary file 4 — Additional file 4. Microglial AhR deficiency alters microglial morphological changes. (A) Representative confocal immunofluorescent images of Iba1 in the corpus callosum of AhRfl/fl mice and CX3CR1-AhR−/− mice after a 5-week cuprizone administration. Scale bar: 40 μm. Representative skeletonized images of microglia in the 4 experimental groups corresponding to the white box. (B) The quantification of Iba1 positive staining in the corpus callosum in (A) (n = 4 mice per group). Data are shown as mean ± SEM and analyzed by two-way ANOVA with Tukey’s multiple comparisons test. (C and D) The quantification of microglial process endpoints and process length per cell in 4 experimental groups (n = 4 mice per group). Data are shown as mean ± SEM and analyzed by two-way ANOVA with Tukey's multiple comparisons test. *p < 0.05, **p < 0.01, ***p < 0.001. [file 12974_2023_2764_MOESM4_ESM.tif]

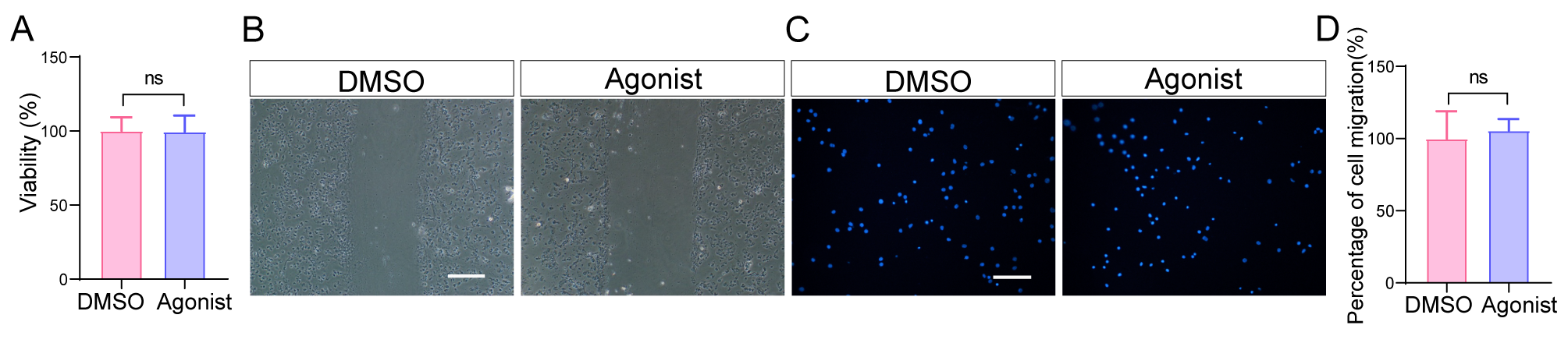

Supplement: Supplementary file 5 — Additional file 5. AhR has no effect on microglia viability and migration in vitro. (A) Viability of primary microglia cultured in vitro and treated with DMSO or AhR agonist I3S was analyzed by CellTiter-Glo assay (n = 3 biological replicates). Data are shown as mean ± SEM and analyzed by unpaired two-tailed t-test. (B) Representative images displaying the wound scratch assay of primary microglia treated with DMSO or AhR agonist I3S. Scale bar: 200 μm. (C) Representative images of transwell assay. Primary microglia migrating to the lower compartment were stained with DAPI. Scale bar: 100 μm. (D) The quantification of migrated primary microglia in (D) (n = 3 biological replicates). Data are shown as mean ± SEM and analyzed by unpaired two-tailed t-test. ns (not significant). [file 12974_2023_2764_MOESM5_ESM.tif]

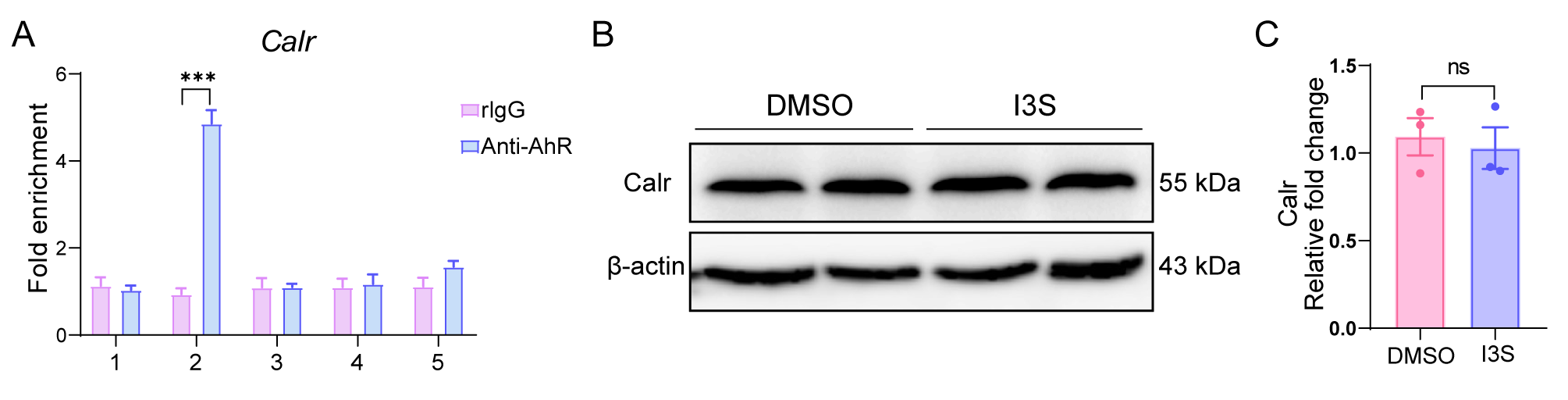

Supplement: Supplementary file 6 — Additional file 6. AhR agonist I3S has no effect on calr expression. (A) ChIP-qPCR analysis was performed in WT microglia to detect the binding of promoter sequences for AhR to Calr (n = 3 biological replicates). The precipitated chromosome segment was PCR-amplified with the use of 5 pairs of specific primers in the Calr promoter. The rlgG is normal rabbit IgG, the homotype control antibody of AhR antibody. Data are shown as mean ± SEM and analyzed by unpaired two-tailed t-test. (B) Western blot analysis of calr protein level in primary microglia treated with DMSO or AhR agonist I3S. 30 μg protein was loaded per well. (C) The quantification of calr protein expression in (I) (n = 3 biological replicates). Data are shown as mean ± SEM and analyzed by unpaired two-tailed t-test. ***p < 0.001, ns (not significant). [file 12974_2023_2764_MOESM6_ESM.tif]

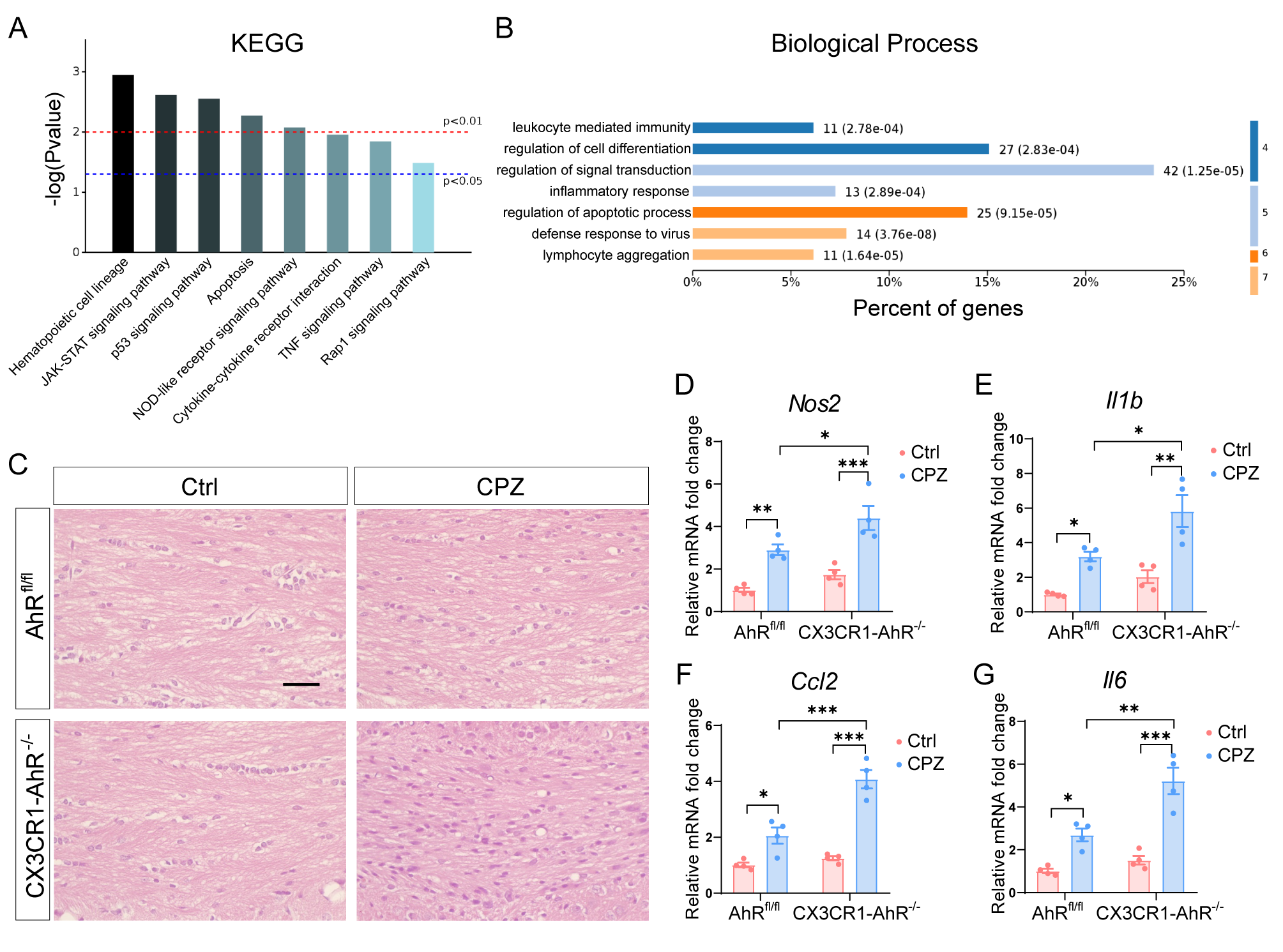

Supplement: Supplementary file 7 — Additional file 7. AhR deficiency in microglia aggravates CNS inflammation. (A) KEGG pathway analysis of 214 upregulated genes unique to CX3CR1-AhR−/− mice after cuprizone administration. (B) GO term enrichment analysis of 214 upregulated genes unique to CX3CR1-AhR−/− mice after cuprizone administration. (C) Representative HE stained images in the corpus callosum of AhRfl/fl mice and CX3CR1-AhR−/− mice in the context of the end of week 6 in the cuprizone model. Scale bar: 50 μm. (D-G) mRNA expressions of Nos2, Il1b, Il6 and Ccl2 in the corpus callosum of AhRfl/fl mice and CX3CR1-AhR−/− mice at the end of week 6 of cuprizone model were evaluated by qRT-PCR (n = 4 mice per group). Data are shown as mean ± SEM and analyzed by two-way ANOVA with Tukey's multiple comparisons test. *p < 0.05, **p < 0.01, ***p < 0.001. [file 12974_2023_2764_MOESM7_ESM.tif]
